# Oxidative cycloaddition of hydroxamic acids with dienes or guaiacols mediated by iodine(III) reagents

**DOI:** 10.3762/bjoc.14.39

**Published:** 2018-02-28

**Authors:** Hisato Shimizu, Akira Yoshimura, Keiichi Noguchi, Victor N Nemykin, Viktor V Zhdankin, Akio Saito

**Affiliations:** 1 Division of Applied Chemistry, Institute of Engineering, Tokyo University of Agriculture and Technology, 2-24-16 Naka-cho, Koganei, Tokyo 184-8588, Japan; 2 Department of Chemistry and Biochemistry, University of Minnesota, Duluth, MN 55812, USA; 3 The Tomsk Polytechnic University, Tomsk 634050, Russia; 4 Instrumentation Analysis Center, Institute of Engineering, Tokyo University of Agriculture and Technology, 2-24-16 Naka-cho, Koganei, Tokyo 184-8588, Japan,; 5 Department of Chemistry, University of Manitoba, Winnipeg, MB R3T 2N2, Canada

**Keywords:** acylnitroso, benzoquinone, cycloaddition, dearomatization, iodine(III)

## Abstract

[Bis(trifluoroacetoxy)iodo]benzene (BTI) and (diacetoxyiodo)benzene (DIB) efficiently promote the formation of acylnitroso species from hydroxamic acids in the presence of various dienes to give the corresponding hetero-Diels–Alder (HDA) adducts in moderate to high yields. The present method could be applied to the HDA reactions of acylnitroso species with *o*-benzoquinones generated by the oxidative dearomatization of guaiacols.

## Introduction

**Scheme 1:**
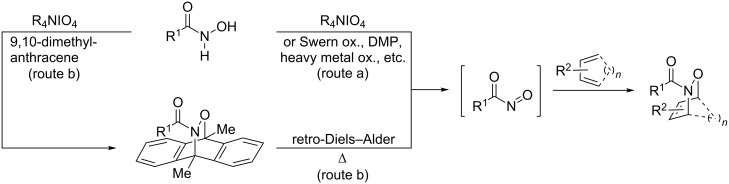
Hetero-Diels–Alder (HDA) reactions of *N*-acylnitroso species.

The hetero-Diels–Alder (HDA) reaction of *N*-acylnitroso species with dienes provides the facile and highly stereoselective synthesis of 1,2-oxazines, which have been widely recognized as useful synthons in the synthesis of biologically active natural products [1]. Generally, in the presence of dienes, acylnitroso species are in situ generated from hydroxamic acids with oxidants due to their instabilities (Scheme 1, route a). Among the oxidants, tetra-*n*-alkylammonium periodates are commonly employed for these HDA reactions, however, the removal of the tetra-*n*-alkylammonium salts, massively generated in these reactions, is often complicated [2–5]. Although the Swern–Moffat oxidation [6], Dess–Martin oxidation [7], and metal-mediated/catalyzed oxidation reactions [8–12] are known as alternative methods [13–14], these methods suffer from a narrow scope and/or low product yields. Furthermore, in the cases where these oxidation methods are inapplicable, a two-step procedure based on the liberation of the acylnitroso species by thermolysis of anthracenyl cycloadducts (route b) is widely used [15–16]. Therefore, there is a need to develop novel oxidation methods of hydroxamic acids for HDA reactions.

As part of our research on the syntheses of heterocycles by iodine(III)-mediated/catalyzed oxidative cycloaddition reactions [17–19], we have found that iodine(III) reagents are effective in the oxidation of N–O bonds of oximes in the cycloaddition reaction of in situ formed nitrile oxides [20–21]. Although Adam and Bottke’s group have demonstrated that (diacetoxyiodo)benzene (DIB) and iodosylbenzene are applicable to the ene reactions of acylnitroso species derived from hydroxamic acids [22], the iodine(III)-mediated oxidative cycloaddition reaction of hydroxamic acids with dienes is still unknown. Herein, we report the HDA reaction of acylnitroso species generated from hydroxamic acids by [bis(trifluoroacetoxy)iodo]benzene (BTI) or DIB. The present method could be applied to HDA reactions with not only simple dienes but also with masked *o*-benzoquinones (MOBs) generated by the oxidative dearomatization of guaiacols.

## Results and Discussion

Our initial studies commenced with the screening of solvents and I(III) reagents for the oxidative cycloaddition reaction of hydroxamic acid **1a** with 1,3-cyclohexadiene (**2a,** Table 1). In the presence of BTI (1.5 equiv), in polar solvents such as methanol, tetrahydrofuran (THF), acetonitrile, and ethyl acetate (Table 1, entries 1–4), or in organochlorine solvents such as chloroform, dichloroethane (DCE), and dichloromethane (DCM, Table 1, entries 6–8), the HDA reactions smoothly proceeded at room temperature within 24 h to give the desired *endo*-cycloadduct **3aa** in 78–98% yield. In particular, the reaction in DCM led to a full conversion to **3aa** within 1 h (Table 1, entry 8) and a reduced amount of BTI (even 1.1 equiv) brought about the similar result (**3aa**: 98%, Table 1, entry 9). Although replacing BTI with DIB or iodosylbenzene (1.5 equiv) afforded the HDA adducts **3aa** in 96 and 90% yields, respectively (Table 1, entries 10 and 11), BTI showed better results (Table 1, entries 8 and 9). On the other hand, the use of IBA-OTf, which was effective in the oxidative cycloaddition reaction of oximes [20–21], gave nitrone-trifluoromethanesulfonic acid (TfOH) complex **4** at 40 °C for 24 h in 85% yield (Table 1, entry 12). Furthermore, when IBA-OTf was employed at lower temperature (−40 °C), the HDA adduct **3aa** was obtained in 80% yield (Table 1, entry 13). These results suggest that the initially formed **3aa** is converted into **4** by TfOH derived from IBA-OTf. To test this hypothesis, the oxazine **3aa** was treated with TfOH in DCM at 40 °C for 18 h and afforded **4** quantitatively. Furthermore, the TfOH-mediated formation of nitrone from such HDA adducts has been reported [23]. It should be mentioned that the structures of **3aa** and **4** were determined by single crystal X-ray analysis [24].

**Table 1:** Screening of I(III) reagents and solvents for HDA reaction of **1a** with **2a**.

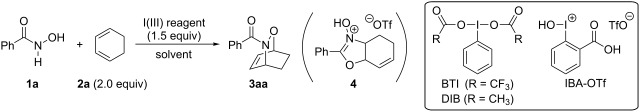					

entry	I(III) reagent	solvent	temp. (°C)	time (h)	**3aa** (%)^a^

1	BTI	MeOH	rt	24	78
2	BTI	THF	rt	24	85
3	BTI	MeCN	rt	24	81
4	BTI	EtOAc	rt	24	89
5	BTI	heptane	rt	24	41
6	BTI	CHCl_3_	rt	24	89
7	BTI	DCE	rt	24	89
8	BTI	DCM	rt	1	98
9	BTI^b^	DCM	rt	1	98
10	DIB	DCM	rt	1	96
11	PhIO	DCM	rt	1	90
12	IBA-OTf	DCM	40	24	0 (**4**: 85^c^)
13	IBA-OTf	DCM	−40	24	80

^a^Isolated yields. ^b^1.1 equiv. ^c^Yield was determined by ^1^H NMR analysis.

Next, we investigated the oxidative cycloaddition reactions of hydroxamic acids **1a**–**c** with various dienes **2** under the optimal conditions (Table 2). Similarly to the benzoyl derivative **1a**, the carbamate analogues **1b** (R^1^ = OBn) and **1c** (R^1^ = O*t*-Bu) reacted with 1,3-cyclohexadiene (**2a**, 2 equiv) in the presence of BTI (1.5 equiv) [25] at room temperature for 1 h to give the corresponding HDA adducts **3ba** and **3ca** in 99 and 85% yields, respectively (Table 2, entries 1–3). However, in the reactions of **1a**–**c** with more reactive dienes such as cyclopentadiene (**2b**) and 9,10-dimethylanthracene (**2c**), DIB (1.1 equiv) showed better results, thereby affording the HDA adducts **3ab** and **3ac**–**3cc** in 69–82% yields (Table 2, entries 4–7). In case of less reactive dienes such as 2,3-dimethyl-1,3-butadiene (**2d**) and isoprene (**2e**), by the use of BTI (1.1 or 1.5 equiv) [25] along with the increase in the amount of diene to 5 equiv, the HDA adducts **3ad**–**3cd** and **3ae**–**3ce** were obtained in moderate to good yields (**3ad**–**3cd**: 55–73%, **3ae**–**3ce**: 49–65%; Table 2, entries 8–13). The HDA adducts **3ae**–**3ce** derived from **2e** were isolated as mixtures of regioisomers (distal:proximal = 2:1 or 1:1 as determined by ^1^H NMR analysis).

**Table 2:** BTI or DIB-mediated oxidative HAD reactions of **1a**–**c** with various dienes.

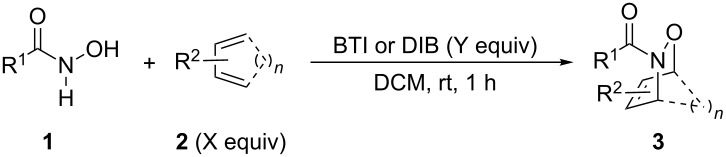					

entry	**3**		X	Y	yield (%)^a^

1	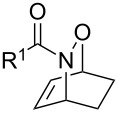	**3aa** (R^1^ = Ph)	2	1.1 (BTI)	98
2		**3ba** (R^1^ = OBn)	2	1.5 (BTI)	99
3		**3ca** (R^1^ = O*t*-Bu)	2	1.5 (BTI)	85
4	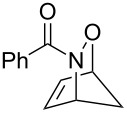	**3ab**	2	1.1 (DIB)	82
5	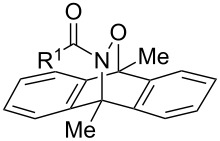	**3ac** (R^1^ = Ph)	2	1.1 (DIB)	69
6		**3bc** (R^1^ = OBn)	2	1.1 (DIB)	78
7		**3cc** (R^1^ = O*t*-Bu)	2	1.1 (DIB)	76
8	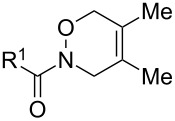	**3ad** (R^1^ = Ph)	5	1.1 (BTI)	61
9		**3bd** (R^1^ = OBn)	5	1.5 (BTI)	73
10		**3cd** (R^1^ = O*t*-Bu)	5	1.5 (BTI)	55
11	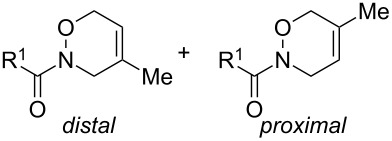	**3ae** (R^1^ = Ph)	5	1.1 (BTI)	49^b^
12		**3be** (R^1^ = OBn)	5	1.5 (BTI)	65^b^
13		**3ce** (R^1^ = O*t*-Bu)	5	1.5 (BTI)	49^c^

^a^Isolated yields. ^b^Distal:proximal = 2:1. ^c^Distal:proximal = 1:1.

**Scheme 2:**
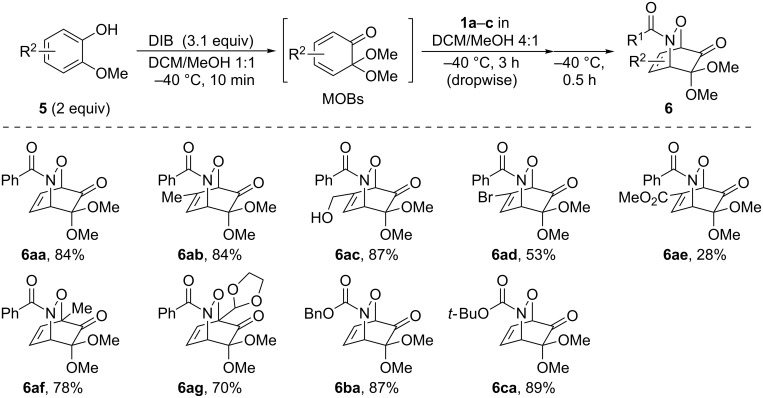
DIB-mediated oxidative HDA reactions of **1a**–**c** with various guaiacols.

As a further application of the iodine(III)-mediated oxidative cycloaddition reactions, the present method was extended to HDA reactions with masked *o*-benzoquinones (MOBs) generated by the oxidative dearomatization [26–29] of guaiacols with methanol (Scheme 2). Liao’s and other groups have developed the DA reactions with MOBs as dienes [30–33], which recently were employed in the HDA reactions of acylnitroso species [34]. However, in the HDA reactions with MOBs, the oxidation of the hydroxamic acids to the acylnitroso species requires other oxidants such as tetra-*n*-alkylammonium periodates. To our delight, the sole use of DIB in a mixed solvent of DCM and methanol gave the *endo*-cycloadduct **6aa** as a single regioisomer in 84% yield starting from hydroxamic acid **1a** and guaiacol (**5a**, Scheme 2). The structure of **6aa** was determined by single crystal X-ray analysis [24]. It should be mentioned that a slow addition of **1a** to the solution of **5a** and DIB over a period of 3 h was essential for an effective formation of **6aa**. Furthermore, this procedure could be applied successfully to the oxidative cycloaddition reaction of **1a** with various guaiacols **5b**–**g** and to the reaction of hydroxamic acids **1b** and **1c** with **5a**. The corresponding products **6ab**–**6ag**, **6ba**, and **6ca** were obtained with complete regioselectivities in 70–89% yields, albeit low to moderate yields in cases of the bromo-substituted **6ad** (53%) and methoxycarbonyl-substituted **6ae** (28%).

In most cases of the attempted reactions of **1a** with simple dienes or guaiacols, benzoyl anhydride was detected as a byproduct. Since benzoyl anhydride is known to be formed via the dimerization of benzoylnitroso compounds [35], these results suggest that acylnitroso species would be generated from **1a** by iodine(III) reagents. Therefore, the high *endo* selectivities of 1,2-oxadines **3aa**–**3ca**, **3ab**, and **6** would depend on the avoidance of electrostatic repulsion between the nitrogen lone pairs of acylnitroso species with π-electrons of the electron-rich dienes in the *exo* transition states (*exo*-lone-pair effect) [36]. Furthermore, the regioselectivities observed for **3ae**, **3be** [12,36], and **6** [34] are in consistency with experimental data as well as the results of FMO and DFT calculations. The decline in regioselectivity of **3ce** may be due to steric repulsion between the sterically hindered *tert*-butyloxycarbonyl group in **1c** with the 2-methyl substituent of isoprene.

## Conclusion

In summary, we demonstrated that BTI and DIB promote the formation of acylnitroso species from hydroxamic acids in the presence of various simple dienes to give the corresponding HDA adducts in moderate to high yields. The present method could be applied to a one-pot reaction involving the generation of MOBs by the dearomatization of guaiacols followed by the HDA reactions of acylnitroso species with MOBs as dienes. Our findings provide an extended scope of dienes for the HDA reactions and HDA reactions of acylnitroso species with MOBs using single oxidants.

## Supporting Information

File 1 Experimental section.

File 2 X-ray structure of **3aa**.

File 3 X-ray structure of **4**.

File 4 X-ray structure of **6aa**.
