# Supplementary material for: Oxidative cycloaddition of hydroxamic acids with dienes or guaiacols mediated by iodine(III) reagents
Source: Beilstein J Org Chem. 2018 Feb 28;14:531–6. doi: 10.3762/bjoc.14.39 (PMC5852453; doi:10.3762/bjoc.14.39)

Supporting Information

for

Oxidative cycloaddition of hydroxamic acids with dienes or guaiacols mediated by iodine(III) reagents

Hisato Shimizu[1], Akira Yoshimura[2,3], Keiichi Noguchi[4], Victor N. Nemykin[5], Viktor V. Zhdankin[2], and Akio Saito*[1]

Address: [1]Division of Applied Chemistry, Institute of Engineering, Tokyo University of Agriculture and Technology, 2-24-16 Naka-cho, Koganei, Tokyo 184-8588, Japan, [2]Department of Chemistry and Biochemistry, University of Minnesota Duluth, MN 55812, USA, [3]The Tomsk Polytechnic University, 634050 Tomsk, Russia, [4]Instrumentation Analysis Center, Institute of Engineering, Tokyo University of Agriculture and Technology, 2-24-16 Naka-cho, Koganei, Tokyo 184-8588, Japan, and [5]Department of Chemistry, University of Manitoba, Winnipeg, MB R3T 2N2, Canada

Email: Akio Saito* - akio-sai@cc.tuat.ac.jp

*Corresponding author

Table of contents

General information..........S1
General procedure for the the oxidative cycloaddition reaction of hydroxamic acids with dienes, and characterization of 1,2-oxazine **3**..........S1
Preparation of nitrone-TfOH complex **4**, and characterization of **4**..........S3
General procedure for the oxidative cycloaddition reaction of hydroxamic acids with guaiacols, and characterization of 1,2-oxazine **6**..........S4
X-Ray crystal structures (ORTEP) of products **3aa**, **4**, and **6aa** (Figure S1)..........S6
^{1}H and ^{13}C NMR spectra of products **3aa-3ca**, **3ab**, **3ca–3cc**, **3ad–3cd**, **3ae–3ce**, **4**, **6aa–6ag**, **6ba**, and **6ca**..........S7

General information

Hydroxamic acid **1a**–**c**, (diacetoxyiodo)benzene (DIB), [bis(trifluoroacetoxy)iodo]benzene (BTI), 1,3-cyclohexadiene, 9,10-dimethylanthracene, 2,3-dimethyl-1,3-butadiene, isoprene, and guaiacols **5a–e** are commercially available. Dichloromethane (DCM) and methanol were purchased "anhydrous" and used without further purification. Cyclopentadiene was prepared by cracking dicyclopentadiene.[1] Guaiacols **5f**[2] and **5g**[3] were prepared by the reported procedures. All reactions were carried out under an argon atmosphere. Column chromatography was performed on silica gel 60N (63–200 μm, neutral, Kanto Kagaku Co., Ltd.) or on alumina (Aluminium Oxides 90 standardized, 63–200 μm, activity II–III acc. to Brockmann, basic, Merck KGaA). ^{1}H and ^{13}C NMR spectra were measured at 300 (or 500) and 75 (or 125) MHz in $CDCl_3$, and the chemical shifts are given in ppm using $CHCl_3$ (7.26 ppm) in $CDCl_3$ for ^{1}H NMR and $CDCl_3$ (77.0 ppm) for ^{13}C NMR as an internal standard, respectively. Splitting patterns of an apparent multiplet associated with an averaged coupling constant were designed as s (singlet), d (doublet), t (triplet), m (multiplet), and br (broadened). Mass spectra and HRMS were recorded on double-focussing magnetic sector by FAB methods.

General procedure for the oxidative cycloaddition reaction of hydroxamic acids with dienes and characterization of 1,2-oxazine 3

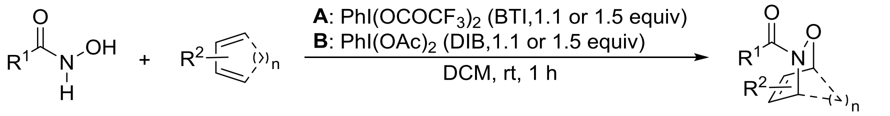

1a: R^{1} = Ph
1b: R^{1} = OBn
1c: R^{1} = O^{t}Bu

2a (1,3-cyclohexadine, 2 equiv)
2b (cyclopentadine, 2 equiv)
2c (9,10-dimethylanthracene, 2 equiv)
2d (2,3-dimethyl-1,3-butadiene, 5 equiv)
2e (isoprene, 5 equiv)

3

To a suspension of hydroxamic acid (**1a**: 54.9 mg, **1b**: 66.9 mg, **1c**: 53.3 mg; 0.40 mmol) and diene (**2a**: 76.2 μL, **2b**: 79.2 μL, **2c**: 165.1 mg; 0.80 mmol, **2d**: 226 μL, **2e**: 241 μL; 2.0 mmol) in DCM (4.5 mL) was added BTI (189 or 258 mg, 0.44 or 0.60 mmol) or DIB (142 or 193 mg, 0.44 or 0.60 mmol) at room temperature. After being stirred at ambient temperature for 1 h, the reaction mixture was quenched with sat. $NaHCO_3$ and sat. $Na_2S_2O_3$, and then extracted with DCM. The organic layer was dried over $MgSO_4$ and concentrated in vacuo to dryness. The residue was purified by preparative thin layer chromatography (PTLC, hexane/EtOAc 3:1 or 1:1) to give **3aa**, **3ab**, **3ad**, **3ae**, **3ba**, **3bd** and **3be**, by column chromatography on alumina (hexane/EtOAc 30:1–5:1) to give **3ac**, **3bc** and **3cc**, or by column chromatography on silica gel (hexane/EtOAc 30:1–5:1) to give **3ca**, **3cd** and **3ce**.

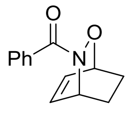

3-Oxa-2-aza-bicyclo[2.2.2]oct-5-en-2-yl(phenyl)methanone (**3aa**): Method **A** (BTI: 1.1 equiv), 84.3 mg (98%). R_f = 0.17 (hexane:EtOAc = 3:1); white solid. ^{1}H NMR (300 MHz, rotameric mixture) δ; 7.61 (br.s, 2H), 7.48-7.26 (m, 3H), 6.63 (br.s, 0.6H), 6.51 (br.s, 1.4H), 5.37 (brs, 0.6H), 4.76 (br.s, 1.4H), 2.42-2.07 (m, 2H), 1.65-1.37 (m, 2H). ^{13}C NMR (75 MHz, rotameric mixture) δ; 168.4, 133.9, 132.9, 131.4, 128.2, 127.6, 71.5, 46.4, 23.0, 20.6. The ^{1}H and ^{13}C NMR spectra of **3aa** were identical to data reported in the literature.[4]

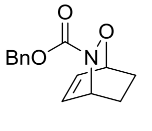

Benzyl 3-oxa-2-aza-bicyclo[2.2.2]oct-5-ene-2-carboxylate (**3ba**): Method **A** (BTI: 1.5 equiv), 97.1 mg (99%). R_f = 0.39 (hexane:EtOAc = 3:1); yellow solid. ^{1}H NMR (300 MHz) δ; 7.29 (br.s, 5H), 6.49 (br.s, 2H), 5.15 (d, J = 12.3 Hz, 1H), 5.07 (d, J = 12.3 Hz, 1H), 4.76 (br.s, 1H), 4.71 (br.s, 1H), 2.18-1.94 (m, 2H), 1.56-1.24 (m, 2H). ^{13}C NMR (75 MHz) δ; 158.1, 135.8, 131.8, 131.5, 128.3, 128.0, 127.9, 70.8, 67.5, 49.9, 23.1, 20.2. The ^{1}H and ^{13}C NMR spectra of **3ba** were identical to data reported in the literature.[5]

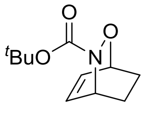

***tert*-Butyl 3-oxa-2-aza-bicyclo[2.2.2]oct-5-ene-2-carboxylate** (**3ca**): Method **A** (BTI: 1.5 equiv), 71.5 mg (85%). R_f = 0.38 (hexane:EtOAc = 3:1); yellow oil. ^{1}H NMR (300 MHz) δ; 6.41 (br.s, 2H), 4.59 (br.s, 2H), 2.10-1.88 (m, 2H), 1.42-1.06 (m, 2H), 1.33 (s, 9H). ^{13}C NMR (75 MHz) δ; 157.5, 131.4, 131.3, 81.1, 70.3, 49.8, 27.7, 23.2, 20.1. The ^{1}H NMR spectra of **3ca** were identical to data reported in the literature.[5]

[1] Moffett, R. B. *Org. Synth., Coll. Vol. IV* **1963**, 238.
[2] Lai, C.-H.; Shen, Y.-L.; Wang, M.-N.; Rao, N. S. K.; Liao. C.-C. *J. Org. Chem.* **2002**, *67*, 6493.
[3] Piel, I.; Steinmetz, M.; Hirano, K.; Fröhlich, R.; Grimme, S.; Glorius, F. *Angew. Chem. Int. Ed.* **2011**, *50*, 4983.
[4] Chaiyaveij, D.; Batsanov, A. S.; Fox, M. A.; Marder, T. B.; Whiting, A. *J. Org. Chem.* **2015**, *80*, 9518.
[5] Chaiyaveij, D.; Cleary, L.; Batsanov, A. S.; Marder, T. B.; Shea, K. J.; Whiting, A. *Org. Lett.* **2011**, *13*, 3442.

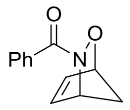

3-Oxa-2-aza-bicyclo[2.2.1]hept-5-en-2-yl(phenyl)methanone (**3ab**): Method **B** (DIB: 1.1 equiv), 66.3 mg (82%). R_f = 0.40 (hexane:EtOAc = 1:1); white solid. ^{1}H NMR (300 MHz, rotameric mixture) δ; 7.84-7.66 (m, 2H), 7.55-7.32 (m, 3H), 6.46 (br.s, 0.8H), 6.35 (br.s, 1.2H), 5.31 (br.s, 1.3H), 5.25 (br.s, 0.7H), 2.09 (d, J = 8.6 Hz, 1H), 1.81 (d, J = 8.6 Hz, 1H). ^{13}C NMR (75 MHz, rotameric mixture) δ; 172.4, 134.3, 132.9, 131.5, 128.6, 128.1, 84.5, 48.0. The ^{1}H NMR spectra of **3ab** were identical to data reported in the literature.[6]

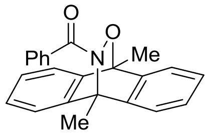

***N*-Benzoyl-9,10-dihydro-9,10-dimethyl-9,10-epoxyiminoanthracene** (**3ac**): Method **B** (DIB: 1.1 equiv), 94.8 mg (69%). R_f = 0.54 (hexane:EtOAc = 3:1); yellow solid. ^{1}H NMR (300 MHz, rotameric mixture) δ; 7.67-7.54 (m, 2H), 7.48-7.26 (m, 11H), 2.81 (s, 2.6H), 2.20 (s, 0.4H), 2.09 (s, 3H). ^{13}C NMR (75 MHz, rotameric mixture) δ; 175.5, 141.6, 141.3, 136.6, 130.5, 128.7, 127.3, 127.1, 121.8, 120.7, 79.8, 63.6, 16.3, 14.7. The ^{1}H NMR spectra of **3ac** were identical to data reported in the literature.[7]

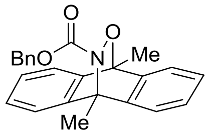

***N*-Benzyloxycarbonyl-9,10-dihydro-9,10-dimethyl-9,10-epoxyiminoanthracene** (**3bc**): Method **B** (DIB: 1.1 equiv), 116.1 mg (78%). R_f = 0.54 (hexane:EtOAc = 3:1); yellow solid. ^{1}H NMR (300 MHz) δ; 7.57-7.41 (m, 2H), 7.41-7.33 (m, 2H), 7.32-7.15 (m, 7H), 6.96 (br.s, 2H), 4.97 (s, 2H), 2.60 (s, 3H), 2.23 (s, 3H). ^{13}C NMR (75 MHz) δ; 159.7, 141.8, 140.5, 136.2, 128.3, 127.6, 127.2, 127.0, 121.5, 120.7, 79.1, 67.0, 63.9, 16.4, 14.8. The ^{1}H NMR spectra of **3bc** were identical to data reported in the literature.[8]

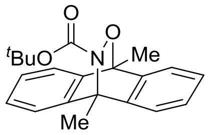

***N-tert*-Butyloxycarbonyl-9,10-dihydro-9,10-dimethyl-9,10-epoxyiminoanthracene** (**3cc**): Method **B** (DIB: 1.1 equiv), 102.9 mg (76%). R_f = 0.58 (hexane:EtOAc = 3:1); yellow solid. ^{1}H NMR (300 MHz) δ; 7.46-7.37 (m, 2H), 7.37-7.30 (m, 2H), 7.25-7.15 (m, 4H), 2.53 (s, 3H), 2.19 (s, 3H), 1.18 (s, 9H). ^{13}C NMR (75 MHz) δ; 159.5, 142.1, 140.6, 127.0, 126.9, 121.4, 120.5, 81.6, 78.5, 64.0, 27.6, 16.6, 14.8. The ^{1}H NMR spectra of **3cc** were identical to data reported in the literature.[9]

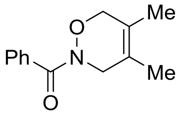

4,5-Dimethyl-3*H*-1,2-oxazin-2(6*H*)-yl(phenyl)methanone (**3ad**): Method **A** (BTI: 1.1 equiv), 53.2 mg (61%). R_f = 0.41 (hexane:EtOAc = 3:1); yellow solid. ^{1}H NMR (300 MHz) δ; 7.72-7.66 (m, 2H), 7.51-7.36 (m, 3H), 4.17 (br.s, 2H), 4.14 (br.s, 2H), 1.70 (s, 3H), 1.58 (s, 3H). ^{13}C NMR (75 MHz) δ; 169.6, 133.7, 130.8, 128.4, 127.9, 122.7, 121.8, 72.6, 46.4, 15.2, 13.6. The ^{1}H and ^{13}C NMR spectra of **3ad** were identical to data reported in the literature.[4]

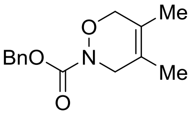

Benzyl 4,5-dimethyl-3*H*-1,2-oxazine-2(6*H*)-carboxylate (**3bd**): Method **A** (BTI: 1.5 equiv), 71.9 mg (73%). R_f = 0.55 (hexane:EtOAc = 3:1); yellow oil. ^{1}H NMR (300 MHz) δ; 7.45-7.19 (m, 5H), 5.17 (s, 2H), 4.17 (br.s, 2H), 3.92 (br.s, 2H), 1.60 (s, 3H), 1.52 (s, 3H). ^{13}C NMR (75 MHz) δ; 155.3, 135.8, 128.2, 127.9, 127.8, 122.8, 121.5, 71.2, 67.2, 48.0, 14.7, 13.3. The ^{1}H and ^{13}C NMR spectra of **3ad** were identical to data reported in the literature.[5]

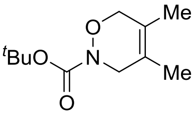

***tert*-Butyl 4,5-dimethyl-3*H*-1,2-oxazine-2(6*H*)-carboxylate** (**3cd**): Method **A** (BTI: 1.5 equiv), 47.2 mg (55%). R_f = 0.62 (hexane:EtOAc = 3:1); yellow oil. ^{1}H NMR (300 MHz) δ; 4.19 (s, 2H), 3.89 (s, 2H), 1.66 (s, 3H), 1.58 (s, 3H), 1.50 (s, 9H). ^{13}C NMR (75 MHz) δ; 154.3, 123.0, 121.8, 81.2, 70.9, 48.2, 28.0, 14.9, 13.5. The ^{1}H and ^{13}C NMR spectra of **3cd** were identical to data reported in the literature.[10]

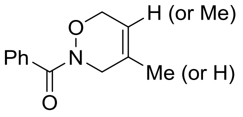

distal (proximal)

4-Methyl-3,6-dihydro-2*H*-1,2-oxazin-2-yl(phenyl)methanone and **5-methyl-3,6-dihydro-2*H*-1,2-oxazin-2-yl(phenyl)methanone** (**3ae**): 39.7 mg (49%, distal:proximal = 2:1). R_f = 0.22 (hexane:EtOAc = 3:1); yellow oil. IR (neat) ν cm^{-1}; 1646. ^{1}H NMR (300 MHz, regioisomeric mixture) δ; 7.75-7.62 (m, 2H), 7.52-7.34 (m, 3H), 5.55 (br.s, 1H), 4.29 (br.s, 2H) , 4.22 (br.s, 2H), 1.78 (s, 2H, dist.), 1.66 (s, 1H, prox.). ^{13}C NMR (75 MHz, regioisomeric mixture) δ; 169.9 (prox.), 169.7(dist.), 133.71 (prox.), 133.68 (dist.), 130.92 (prox.), 130.90 (dist.), 130.2, 128.4, 127.99 (prox.), 127.97 (dist.), 117.5 (dist.), 116.2 (prox.), 72.6 (prox.), 69.5 (dist.), 46.5 (dist.), 42.9 (prox.), 19.7 (dist.), 18.1 (prox.). FAB-LM m/z: 204 (M^{+}+H^{+}). FAB-HM Calcd for $C_{12}H_{14}NO_2$ (M^{+}+H^{+}): 204.1025; found: 204.1007. The regiochemistry of **3ae** was determined by the comparison with the ^{1}H and ^{13}C NMR spectra of **3be**.

---

[6] Dao, L. H.; Dust, J. M.; Mackay, D.; Watson, K. N. *Can. J. Chem.* **1979**, *57*, 1712.
[7] Kirby, G. W.; Sweeny, J. G. *J. Chem. Soc., Perkin Trans 1* **1981**, 3250.
[8] Kirby, G. W.; McGuigan, H.; Mackinnon, J. W. M.; Mclean, D.; Sharma, R. P. *J. Chem. Soc. Perkin Trans. 1* **1985**, 1437.
[9] Jenkins, N. E.; Ware Jr., R. W.; Atkinson, R. N.; King, S. B. *Synth. Commun.* **2000**, *30*, 947.
[10] Flower, K. R.; Lightfoot, A. P.; Wanc, H.; Whiting, A. *J. Chem. Soc., Perkin Trans 1* **2002**, 2058.

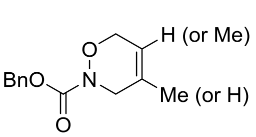

distal (proximal)

Benzyl 4-methyl-3*H*-1,2-oxazine-2(6*H*)-carboxylate and **benzyl 5-methyl-3*H*-1,2-oxazine-2(6*H*)-carboxylate** (**3be**): 60.7 mg (65%, distal:proximal = 2:1). R_f = 0.49 (hexane:EtOAc = 3:1); yellow oil. ^{1}H NMR (300 MHz, regioisomeric mixture) δ; 7.48-7.28 (m, 5H), 5.53 (s, 1H), 5.23 (br.s, 2H), 4.39 (br.s, 1.33H, dist.), 4.29 (br.s, 0.67H, prox.), 4.12 (br.s, 0.67H, prox.), 4.03 (br.s, 1.33H, dist.), 1.73 (s, 2H, dist.), 1.66 (s, 1H, prox.). ^{13}C NMR (75 MHz, regioisomeric mixture) δ; 155.5 (prox.), 155.4 (dist.), 135.9, 131.4 (prox.), 129.9 (dist.), 128.4, 128.1, 127.9, 117.8 (dist.), 116.1 (prox.), 71.3 (prox.), 68.2 (dist.), 67.4, 48.2 (dist.), 44.4 (prox.), 19.3 (dist.), 17.8 (prox.). The ^{1}H and ^{13}C NMR spectra of **3be** were identical to data reported in the literature.[11]

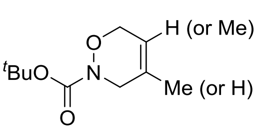

distal (proximal)

***tert*-Butyl 4-methyl-3*H*-1,2-oxazine-2(6*H*)-carboxylate** and ***tert*-butyl 5-methyl-3*H*-1,2-oxazine-2(6*H*)-carboxylate** (**3ce**): 38.7 mg (49%, distal:proximal = 1:1). R_f = 0.63 (hexane: EtOAc = 3:1); yellow oil. ^{1}H NMR (300 MHz, regioisomeric mixture) δ; 5.53 (br.s, 1H), 4.35 (br.s, 1H, dist.), 4.25 (br.s, 1H, prox.), 4.03 (br.s, 1H, prox.), 3.94 (br.s, 1H, dist.), 1.74 (s, 1.5H, dist.), 1.67 (s, 1.5H, prox.), 1.50 (s, 9H). ^{13}C NMR (75 MHz, regioisomeric mixture) δ; 154.9 (prox.), 154.8 (dist.), 131.3 (prox.), 130.1 (dist.), 117.9 (dist.), 116.3 (prox.), 81.1, 71.8 (prox.), 67.6 (dist.), 48.2 (dist.), 44.4 (prox.), 27.9, 19.4 (dist.), 17.9 (prox.). The ^{1}H and ^{13}C NMR spectra of **3ce** were identical to data reported in the literature.[11]

Preparation of nitrone-TfOH complex 4 and characterization

Preparation of 4 from 1a

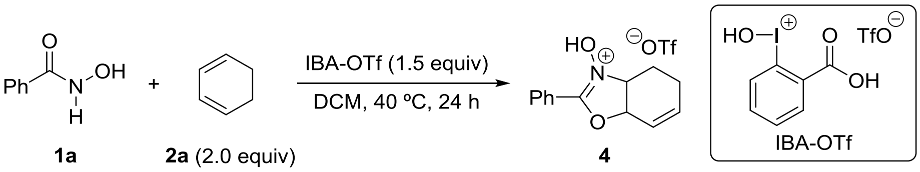

1a **2a** (2.0 equiv) **4**

To a suspension of hydroxamic acid **1a** (54.9 mg, 0.40 mmol) and 1,3-cyclohexadiene (**2a**, 76.2 µL, 0.80 mmol) in DCM (4.5 mL) was added IBA-OTf[12] (247.9 mg, 0.60 mmol) at room temperature. After being stirred at 40 °C for 24 h, the solvent was removed in vacuo. The product **4** was obtained in 85% yield, which was determined by NMR analysis of the crude reaction mixture.

Preparation of 4 from 3aa

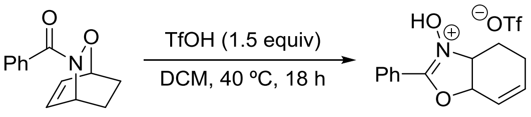

3aa **4**

To a solution of oxazine **3aa** (86.1 mg, 0.40 mmol) in DCM (4.5 mL) was added TfOH (67.8 µL, 0.60 mmol) at room temperature. After being stirred at 40 °C for 18 h, the solvent was removed in vacuo. NMR analysis of the crude reaction mixture showed that oxazine **3aa** was converted to **4** quantitatively. The product **4** was obtained as a colorless crystal by recrystallization (hexane/CH_2Cl_2/EtOAc 3:2:1).

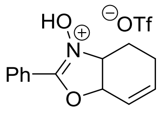

3a,4,5,7a-Tetrahydro-2-phenylbenzo[*d*]oxazole-*N*-oxide–trifluoromethanesulfonic acid complex (**4**): Mp 88-89 °C. IR (KBr) ν cm^{-1}; 1640, 944. ^{1}H NMR (500 MHz) δ; 11.79 (br.s, 1H), 8.25 (d, *J* = 7.8 Hz, 2H), 7.78 (t, *J* = 7.8 Hz, 1H), 7.58 (t, *J* = 7.8 Hz, 2H), 6.44 (dd, *J* = 9.7, 3.5 Hz, 1H), 5.96 (dd, *J* = 9.7, 3.5 Hz, 1H), 5.69 (dd, *J* = 9.7, 2.3 Hz, 1H), 5.11-5.01 (m, 1H), 2.39-2.20 (m, 2H), 2.20-2.05 (m, 2H). ^{13}C NMR (125 MHz) δ; 164.6, 138.6, 136.9, 131.0, 129.3, 120.0 (q, *J* = 319.1 Hz), 119.0, 118.9, 78.3, 62.0, 21.2, 19.8. FAB-LM m/z: 216 (M^+–OTf). FAB-HM Calcd for $C_{13}H_{14}NO_2$ (M^+–OTf): 216.1025; found: 216.1015.

[11] Tolman, V.; Hanuš, J.; Sedmer, P. *Collect. Czech. Chem. Commun.* **1999**, *64*, 696.
[12] Yoshimura, A.; Nguyen, K. C.; Klasen, S.; Saito, A.; Nemykin, V. N.; Zhdankin, V. V. *Chem. Commun.* **2015**, *51*, 7835.

General procedure for the oxidative cycloaddition reaction of hydroxamic acids with guaiacols, and characterization of 1,2-oxazine 6

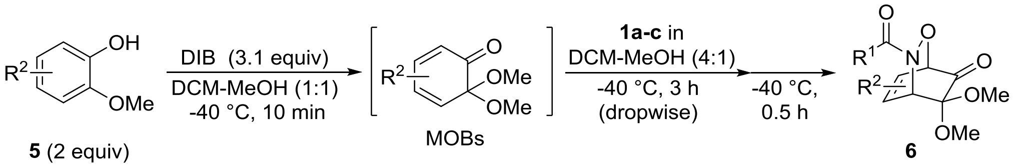

To a solution of the masked *o*-benzoquinones, which was in situ prepared by the teatment of guaiacols **5** (0.80 mmol) with DIB (399 mg, 1.24 mmol) for 10 min at −40 °C in the mixed solvent of MeOH (3.0 mL) and DCM (3.0 mL), was added dropwise a solution of hydroxamic acid (**1a**: 54.9 mg, **1b**: 66.9 mg, **1c**: 53.3 mg; 0.40 mmol) in the mixed solvent of MeOH (0.5 mL) and DCM (2.0 mL) at the same temperature over 3 h. After being stirred at the same temperature for 0.5 h, the reaction mixture was allowed to warm to room temperature, and then concentrated in vacuo to dryness. The residue was purified by medium pressure liquid chromatography (MPLC, hexane/EtOAc 1:4, flow rate 20 mL/min) to give **6aa**–**6ag** and **6ba**, or by PTLC (hexane/EtOAc 1:1) to give **5ca**.

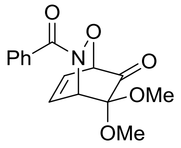

(1*R,4*S**)-3-Benzoyl-5,5-dimethoxy-2-oxa-3-azabicyclo[2.2.2]oct-7-en-6-one** (**6aa**): 97.0 mg (84%). R_f = 0.22 (hexane:EtOAc = 3:1); white solid; mp 124-125 ºC. IR (KBr) ν cm^{-1}; 1758, 1655, 1145, 1104. ^{1}H NMR (300 MHz) δ; 7.79-7.71 (m, 2H), 7.54-7.46 (m, 1H), 7.45-7.36 (m, 2H), 6.97 (dd, *J* = 8.1, 6.0 Hz, 1H), 6.48 (ddd, *J* = 8.1, 6.0, 1.8 Hz, 1H), 5.65 (br.s, 1H), 4.72 (d, *J* = 6.0 Hz, 1H), 3.50 (s, 3H), 3.46 (s, 3H). ^{13}C NMR (75 MHz) δ; 193.9, 171.2, 138.2, 132.8, 131.5, 128.9, 127.8, 126.5, 91.8, 78.5, 54.7, 50.8, 50.6. FAB-LM m/z: 290 (M$^+$+H$^+$). FAB-HM Calcd for $C_{15}H_{16}NO_5$ (M$^+$+H$^+$): 290.1028; found: 290.1007.

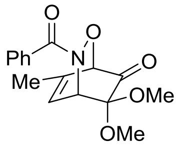

(1*R,4*S**)-3-Benzoyl-5,5-dimethoxy-7-methyl-2-oxa-3-azabicyclo[2.2.2]oct-7-en-6-one** (**6ab**): 101.8 mg (84%). R_f = 0.29 (hexane:EtOAc = 3:1); white solid; mp 110-112 ºC. IR (KBr) ν cm^{-1}; 1751, 1642, 1151, 1098. ^{1}H NMR (300 MHz) δ; 7.74 (d, *J* = 7.3 Hz, 2H), 7.49 (t, *J* =7.1 Hz, 1H), 7.41 (dd, *J* = 7.1, 7.3 Hz, 2H), 6.55 (br.s, 1H), 5.55 (br.s, 1H), 4.54 (s, 1H), 3.53 (s, 3H), 3.48 (s, 3H), 1.94 (s, 3H). ^{13}C NMR (75 MHz) δ; 194.7, 170.4, 137.2, 133.1, 131.6, 129.0, 128.0, 92.2, 83.0, 54.2, 51.0, 50.8, 18.4. FAB-LM m/z: 304 (M$^+$+H$^+$). FAB-HM Calcd for $C_{15}H_{16}NO_5$ (M$^+$+H$^+$): 304.1185; found: 304.1213.

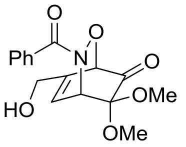

(1*R,4*S**)-3-Benzoyl-5,5-dimethoxy-7-(hydroxymethyl)-2-oxa-3-azabicyclo[2.2.2]oct-7-en-6-one** (**6ac**): 111.1 mg (87%). R_f = 0.19 (hexane:EtOAc = 1:1); yellow oil. IR (neat) ν cm^{-1}; 1759, 1632, 1138, 1071. ^{1}H NMR (300 MHz) δ; 7.73 (d, *J* = 7.0 Hz, 2H), 7.58-7. 34 (m, 3H), 6.75 (br.s, 1H), 5.60 (br.s, 1H), 4.75 (br.s, 1H), 4.27 (s, 2H), 3.48 (s, 3H), 3.43 (s, 3H), 2.22 (br.s, 1H). ^{13}C NMR (75 MHz) δ; 194.4, 170.4, 140.6, 132.8, 131.7, 128.9, 128.1, 92.4, 79.6, 60.7, 54.6, 51.1, 50.8. FAB-LM m/z: 320 (M$^+$+H$^+$). FAB-HM Calcd for $C_{16}H_{18}NO_6$ (M$^+$+H$^+$): 320.1134; found: 320.1155.

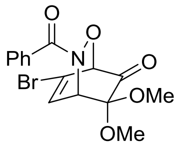

(1*R,4*S**)-3-Benzoyl-7-bromo-5,5-dimethoxy-2-oxa-3-azabicyclo[2.2.2]oct-7-en-6-one** (**6ad**): 77.5 mg (53%). R_f = 0.26 (hexane:EtOAc = 3:1); white solid; mp 128-130 ºC. IR (KBr) ν cm^{-1}; 1759, 1665, 1141, 1097, 717, 693. ^{1}H NMR (300 MHz) δ; 7.80-7.73 (m, 2H), 7.57-7.49 (m, 2H), 7.48-7.38 (m, 2H), 7.10 (dd, *J* = 6.8, 2.2 Hz, 1H), 5.71 (d, *J* = 6.8 Hz, 1H), 4.76 (d, *J* = 2.2 Hz, 1H), 3.47 (s, 6H). ^{13}C NMR (75 MHz) δ; 191.9, 171.4, 137.2, 132.4, 132.1, 129.3, 128.1, 115.8, 92.0, 84.8, 56.0, 51.3, 50.9. FAB-LM m/z: 368 (M$^+$+H$^+$). FAB-HM Calcd for $C_{16}H_{18}NO_6$ (M$^+$+H$^+$): $C_{15}H_{15}BrNO_5$ (M$^+$+H$^+$): 368.0134; found: 368.0163.

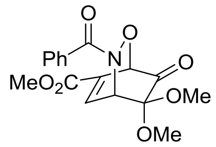

(1*R,4*S**)-3-Benzoyl-5,5-dimethoxy-7-(methoxycarbonyl)-2-oxa-3-azabicyclo[2.2.2]oct-7-en-6-one** (**6ae**): 37.0 mg (28%). R_f = 0.18 (hexane:EtOAc = 3:1); white solid; mp 137-138 ºC. IR (KBr) ν cm^{-1}; 1761, 1718, 1697. ^{1}H NMR (300 MHz) δ; 7.80-7.73 (m, 2H), 7.72 (dd *J* = 6.4, 1.5 Hz, 1H), 7.55-7.47 (m, 1H), 7.44-7.37 (m, 2H), 5.83 (d, *J* = 6.4 Hz, 1H), 5.21 (d, *J* = 1.5 Hz, 1H), 3.80 (s, 3H), 3.49 (s, 3H), 3.45 (s, 3H). ^{13}C NMR (75 MHz) δ; 193.2, 171.5, 161.9, 144.6, 132.4, 132.2, 130.8, 129.5, 128.1, 92.0, 77.7, 54.4, 52.6, 51.4, 50.8. FAB-LM m/z: 348 (M$^+$+H$^+$). FAB-HM Calcd for $C_{16}H_{18}NO_6$ (M$^+$+H$^+$): $C_{15}H_{15}BrNO_5$ (M$^+$+H$^+$): $C_{17}H_{18}NO_7$ (M$^+$+H$^+$): 348.1083; found: 348.1078.

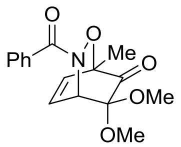

(1*R,4*S**)-3-Benzoyl-5,5-dimethoxy-1-methyl-2-oxa-3-azabicyclo[2.2.2]oct-7-en-6-one** (**6af**): 97.6 mg (78%). R_f = 0.26 (hexane:EtOAc = 3:1); yellow oil. IR (neat) ν cm^{-1}; 1755, 1650, 1070, 1038. ^{1}H NMR (300 MHz) δ; 7.76 (d, *J* = 7.3 Hz, 2H), 7.48 (t, *J* = 7.2, 1H), 7.39 (dd, 7.3, 7.2 Hz, 2H), 6.96-6.90 (m, 1H), 6.18 (dd, J = 8.1, 1.7 Hz, 1H), 5.63 (br.s, 1H), 3.48 (s, 3H), 3.45 (s, 3H), 1.46 (s, 3H). ^{13}C NMR (75 MHz) δ; 194.6, 170.6, 137.6, 132.9, 131.5, 131.0, 129.1, 127.9, 91.8, 82.6, 54.5, 51.0, 50.7, 14.3. FAB-LM m/z: 304 (M^++H^+). FAB-HM Calcd for $C_{15}H_{16}NO_5$ (M^++H^+): 304.1185; found: 304.1173.

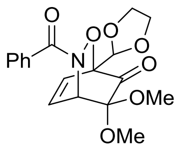

(1*R,4*S**)-3-Benzoyl-5,5-dimethoxy-1-(1,3-dioxolan-2-yl)-2-oxa-3-azabicyclo[2.2.2]oct-7-en-6-one** (**6ag**): 101.8 mg (70%). R_f = 0.12 (hexane:EtOAc = 3:1); white solid; mp 138-140 ºC. IR (KBr) ν cm^{-1}; 1759, 1656, 1145, 1082. ^{1}H NMR (300 MHz) δ; 7.80-7.70 (m, 2H), 7.54-7.31 (m, 3H), 6.99 (br.s, 1H), 6.38 (dd, *J* = 8.1, 1.8 Hz, 1H), 5.60 (br.s, 1H), 5.48 (s, 1H), 4.05-3.71 (m, 3H), 3.67-3.54 (m, 1H) , 3.48 (s, 3H), 3.45 (s, 3H). ^{13}C NMR (75 MHz) δ; 193.2, 172.1, 138.8, 133.1, 131.6, 129.1, 127.8, 50.9, 126.7, 99.5, 92.2, 84.3, 65.9, 65.5, 54.9, 51.1, 50.9. FAB-LM m/z: 362 (M^++H^+). FAB-HM Calcd for $C_{18}H_{20}NO_7$ (M^++H^+): 362.1240; found: 362.1278.

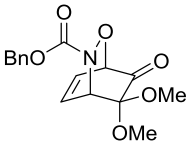

(1*R,4*S**)-3-Benzyloxycarbonyl-5,5-dimethoxy-2-oxa-3-azabicyclo[2.2.2]oct-7-en-6-one** (**6ba**): 111.5 mg (87%). R_f = 0.32 (hexane:EtOAc = 3:1); yellow oil. IR (neat) ν cm^{-1}; 1755, 1712, 1104, 1053. ^{1}H NMR (300 MHz) δ; 7.44-7.34 (m, 5H), 6.80 (dd, *J* = 8.2, 1.6 Hz, 1H), 6.51 (ddd, *J* = 8.2, 6.0, 1.6 Hz, 1H), 5.30-5.12 (m, 3H), 4.76 (dd, *J* = 6.0, 1.6 Hz, 1H), 3.44 (s, 3H), 3.43 (s, 3H). ^{13}C NMR (75 MHz) δ; 193.7, 157.8, 136.9, 135.2, 128.54, 128.48, 128.2, 126.8, 91.4, 78.0, 68.4, 57.4, 50.9, 50.6. FAB-LM m/z: 320 (M^++H^+). FAB-HM Calcd for $C_{16}H_{18}NO_6$ (M^++H^+): 320.1134; found: 320.1155.

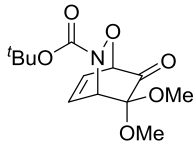

(1*R,4*S**)-3-*tert*-Butyloxycarbonyl-5,5-dimethoxy-2-oxa-3-azabicyclo[2.2.2]oct-7-en-6-one** (**6ca**): 101.3 mg (89%). R_f = 0.69 (hexane:EtOAc = 1:1); white solid; mp 102-105 ºC. IR (KBr) ν cm^{-1}; 1757, 1699. ^{1}H NMR (300 MHz) δ; 6.81 (ddd, *J* = 8.2, 6.0, 1.5, 1H), 6.52 (ddd, *J* = 8.2, 6.0, 2.0 Hz, 1H), 5.15 (dd, J = 6.0, 2.0 Hz, 1H), 4.74 (dd, *J* = 6.0, 1.5 Hz, 1H), 3.46 (s, 3H), 3.44 (s, 3H), 1.47 (s, 9H). ^{13}C NMR (75 MHz) δ; 193.9, 157.0, 136.8, 126.6, 91.4, 83.0, 77.7, 57.5, 50.8, 50.6, 27.8. FAB-LM m/z: 286 (M^++H^+). FAB-HM Calcd for $C_{13}H_{20}NO_6$ (M^++H^+): 286.1291; found: 286.1303.

X-Ray crystal structures (ORTEP) of products 3aa, 4, and 6aa

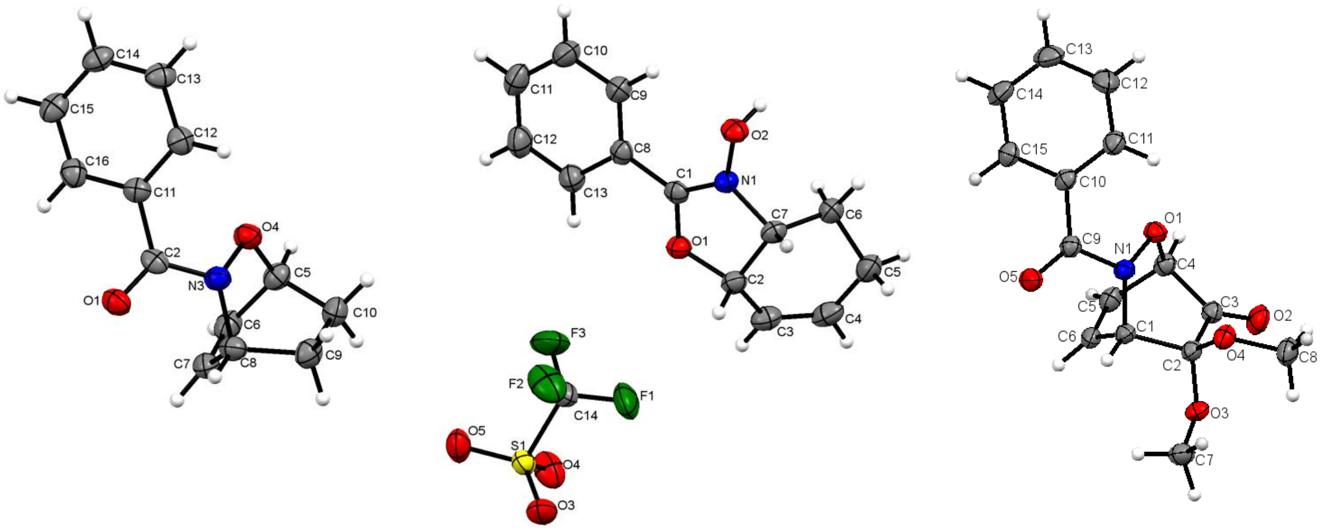

Figure S1: X-Ray crystal structure of **3aa** (left), **4** (center) and **6aa** (right)

^{1}H and ^{13}C NMR spectra of products

^{1}H NMR of **3aa**

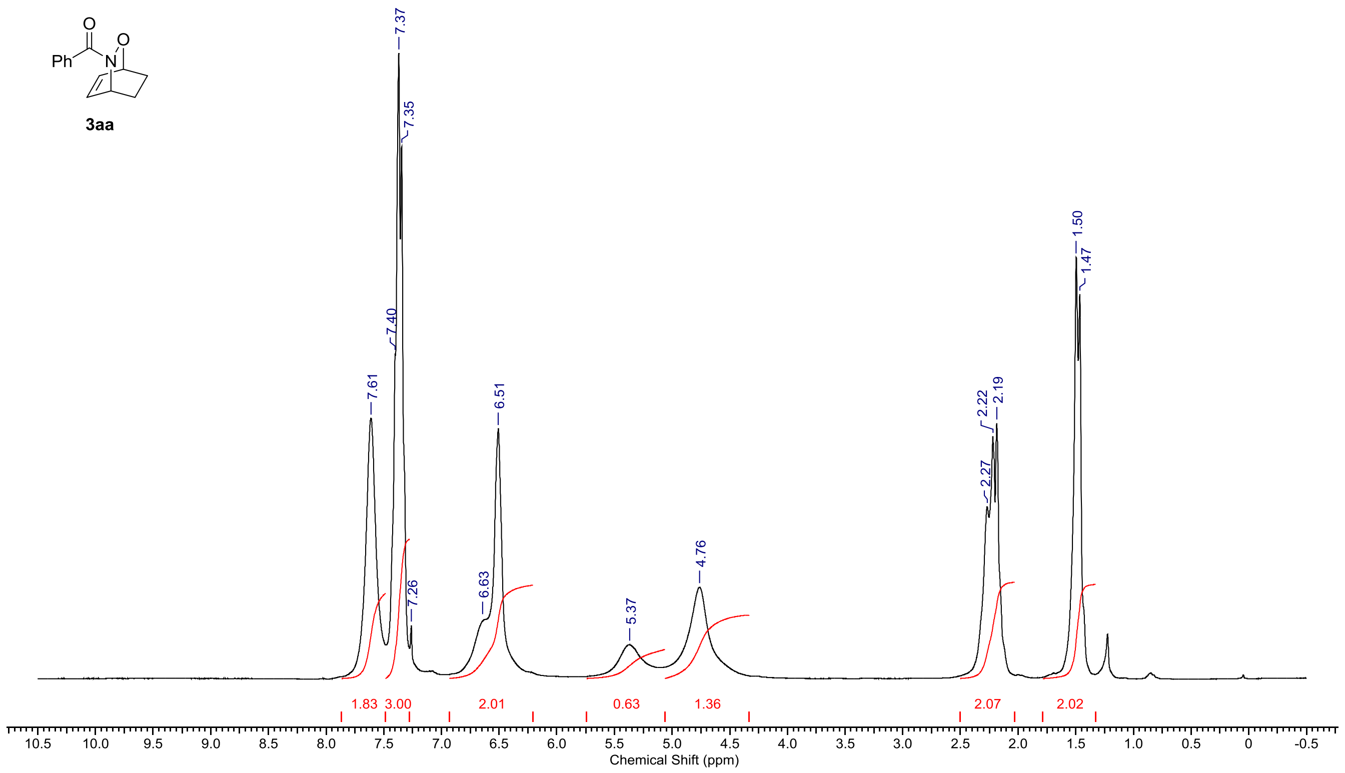

^{13}C NMR of **3aa**

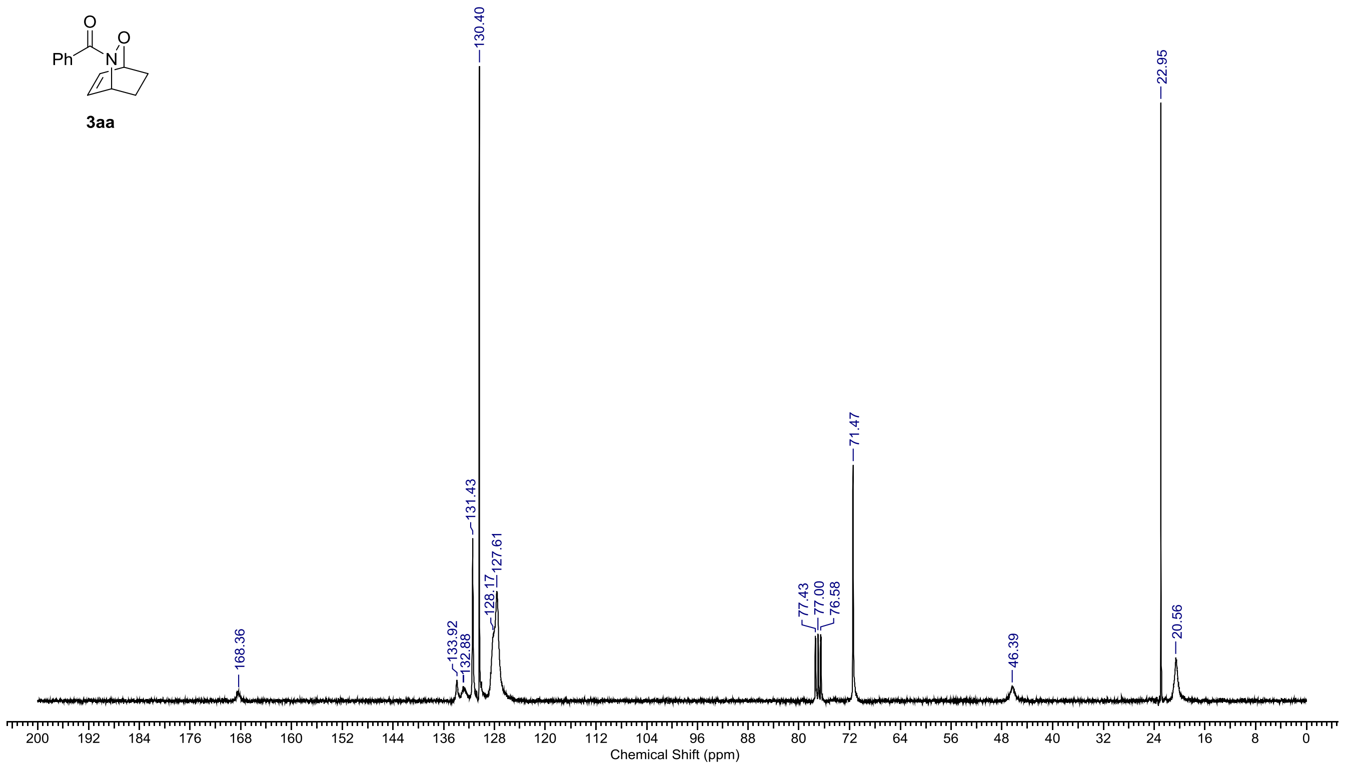

^{1}H NMR of **3ba**

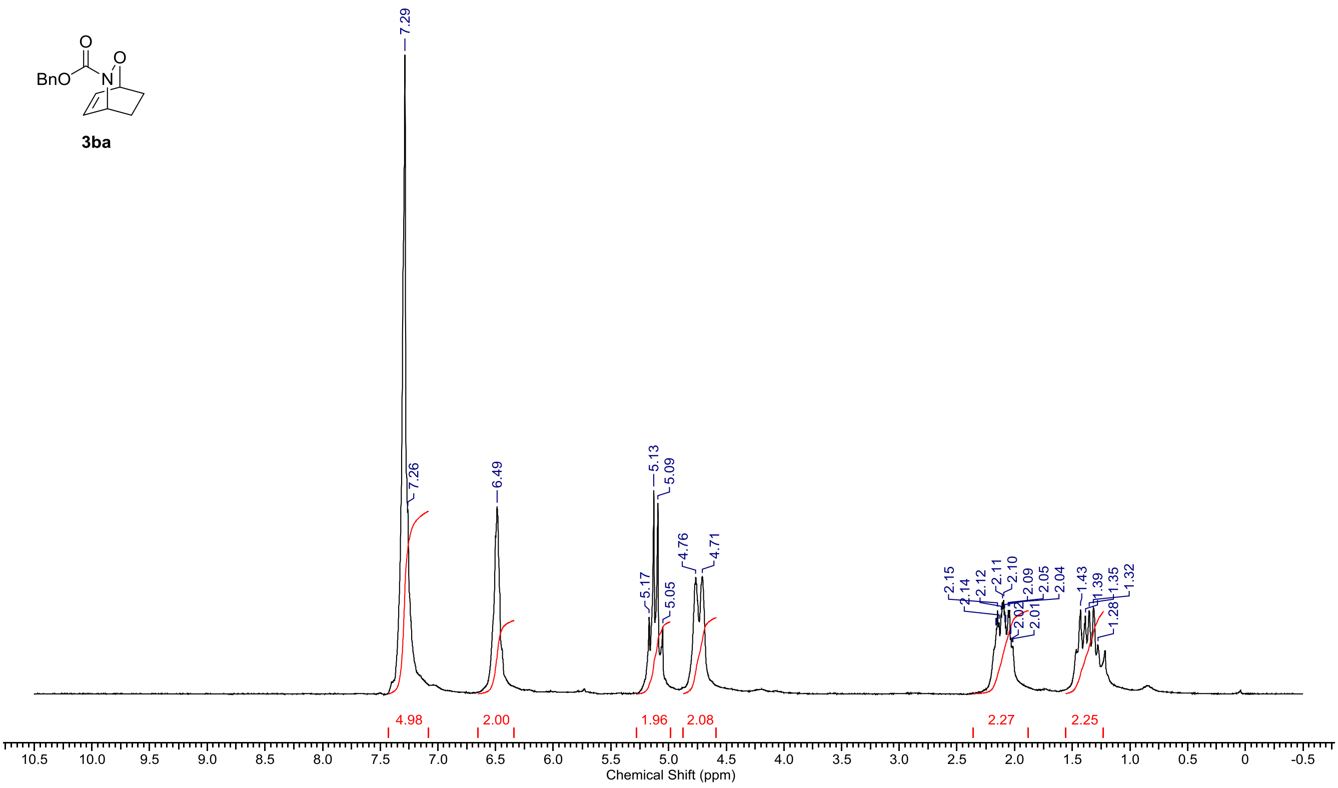

^{13}C NMR of **3ba**

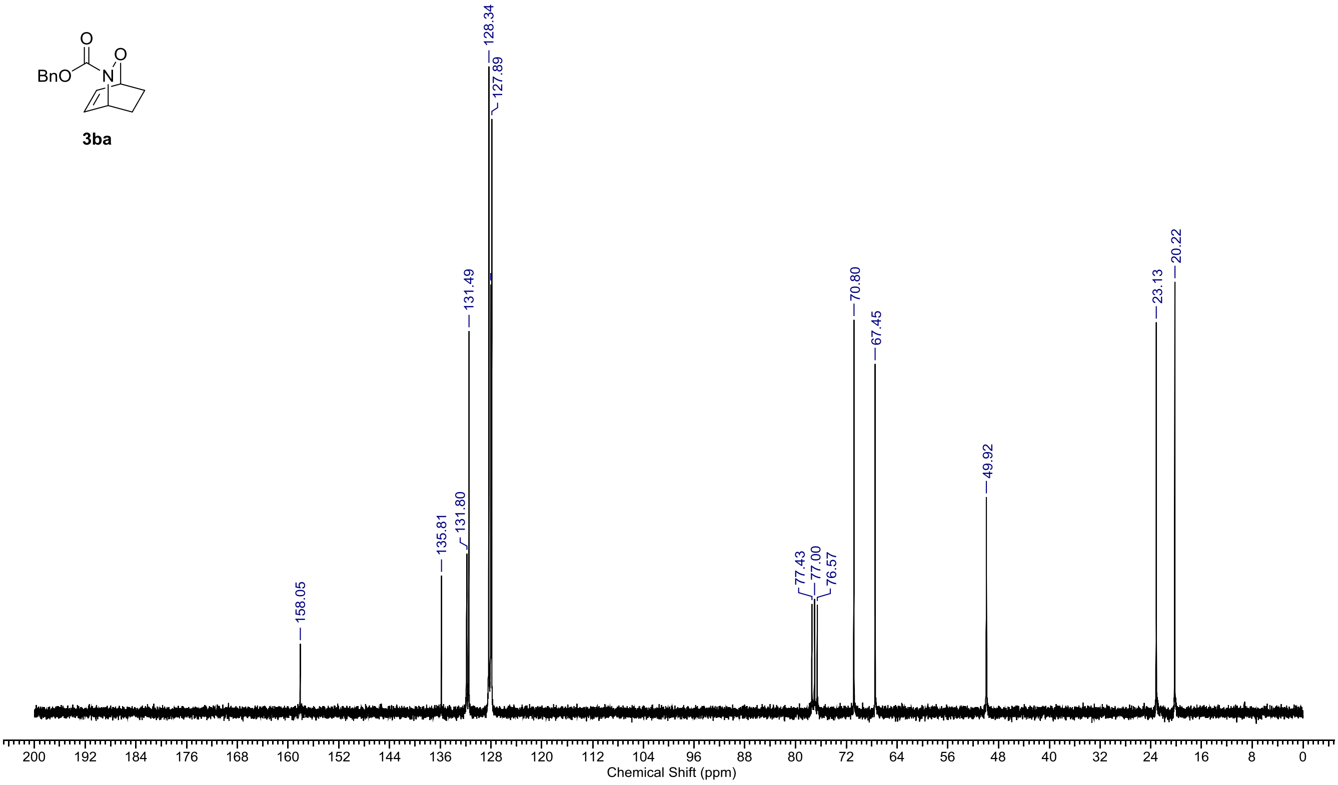

^{1}H NMR of **3ca**

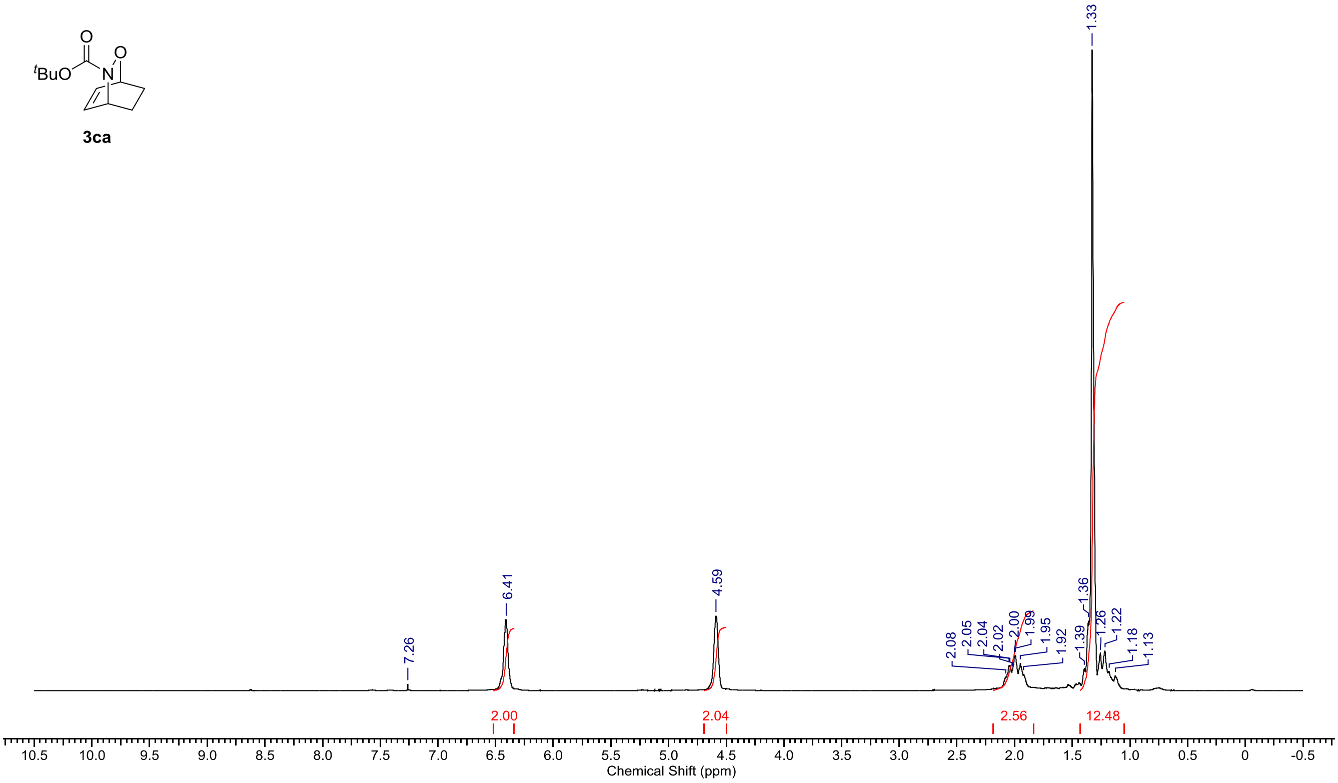

3ca

^{13}C NMR of **3ca**

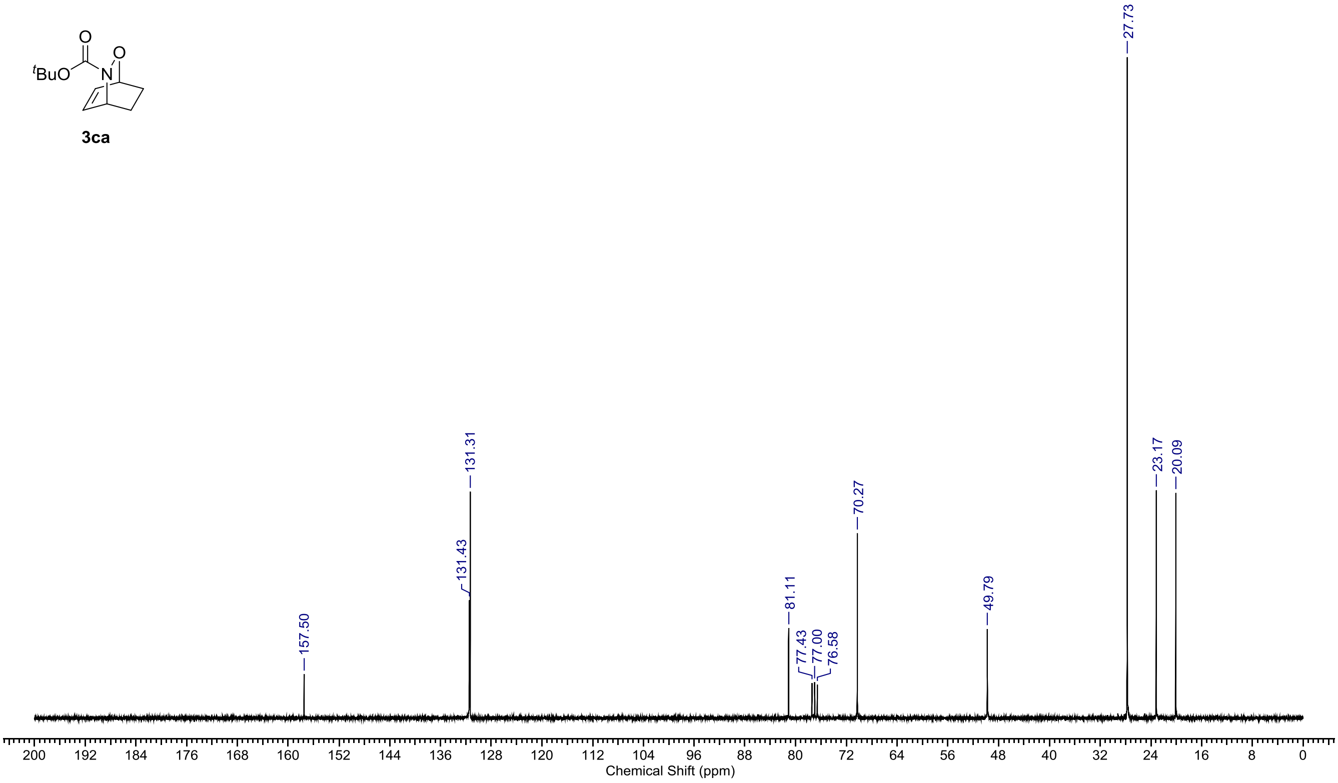

3ca

^{1}H NMR of **3ab**

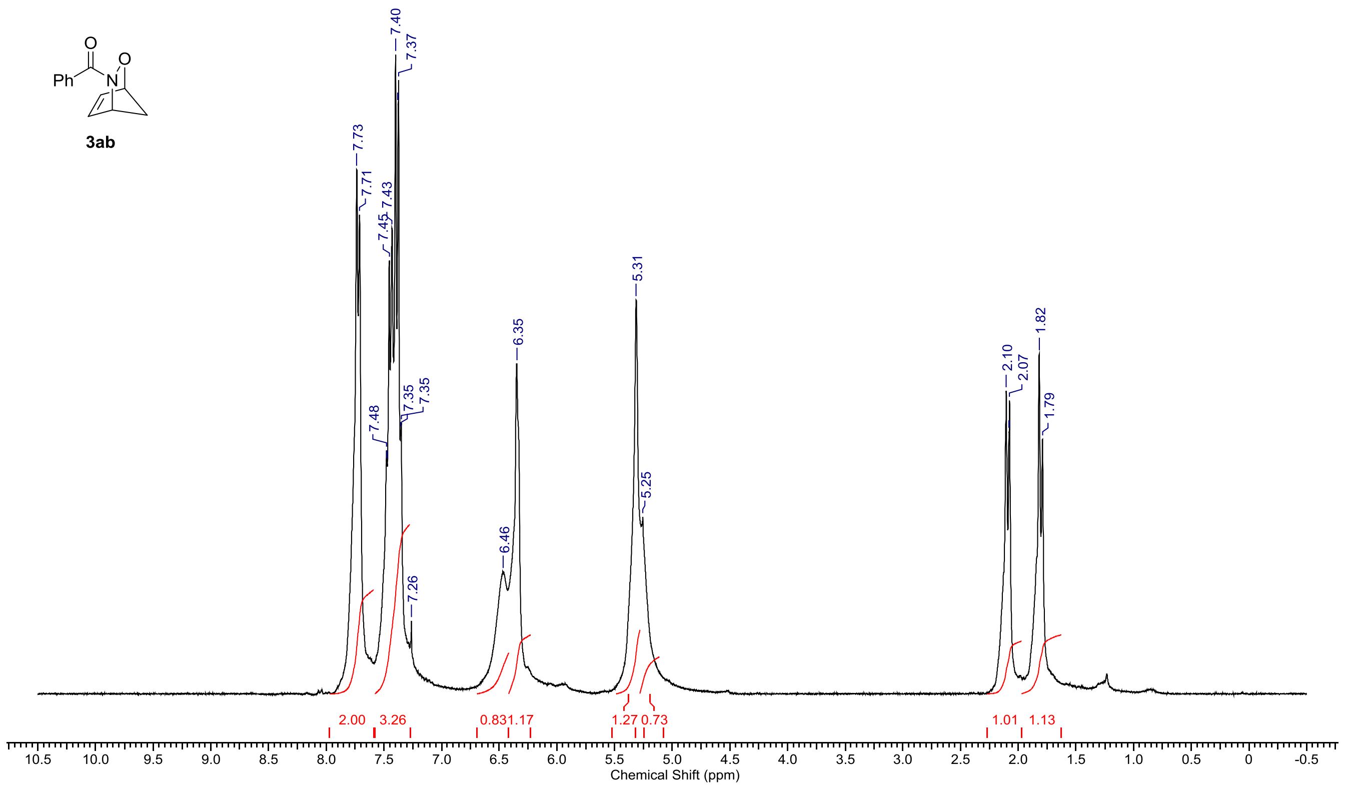

^{13}C NMR of **3ab**

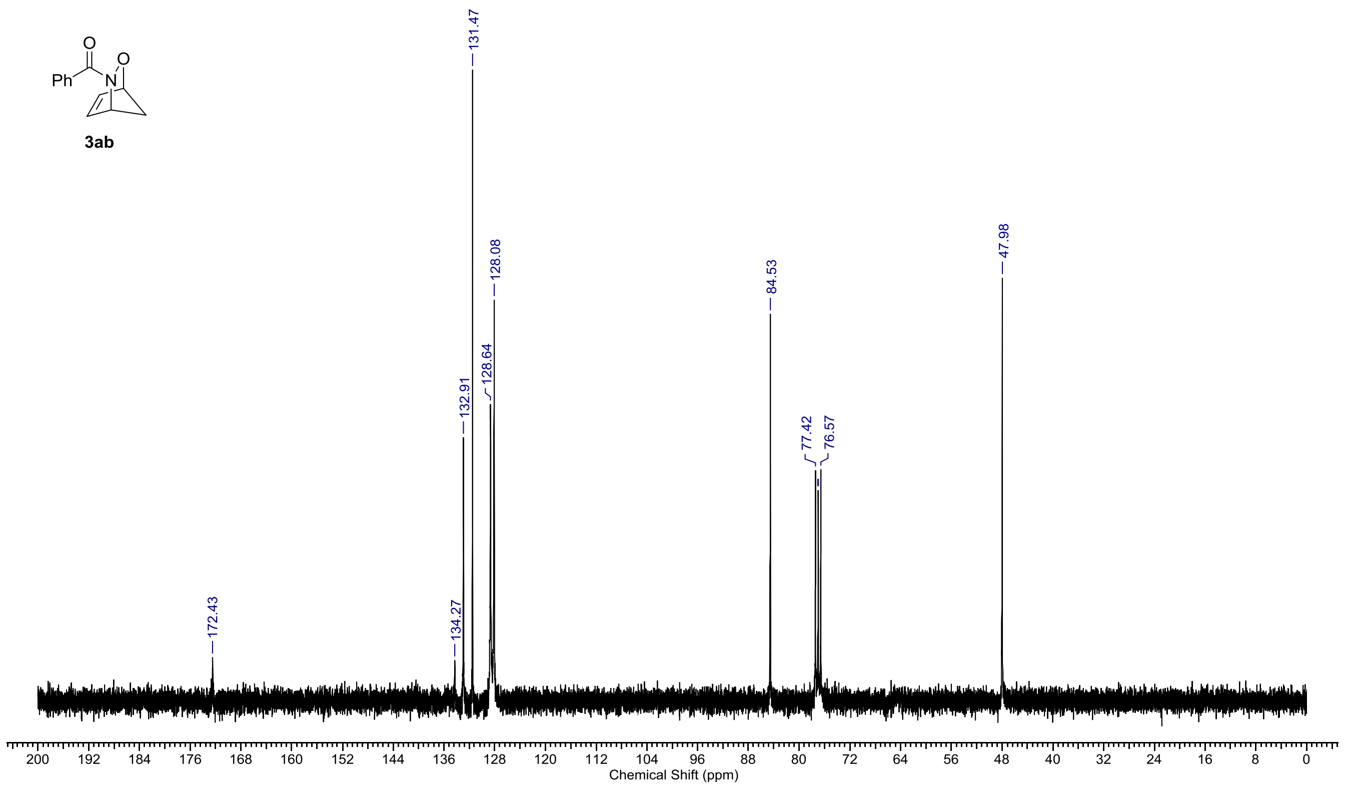

^{1}H NMR of **3ac**

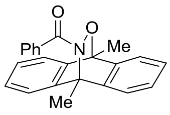

3ac

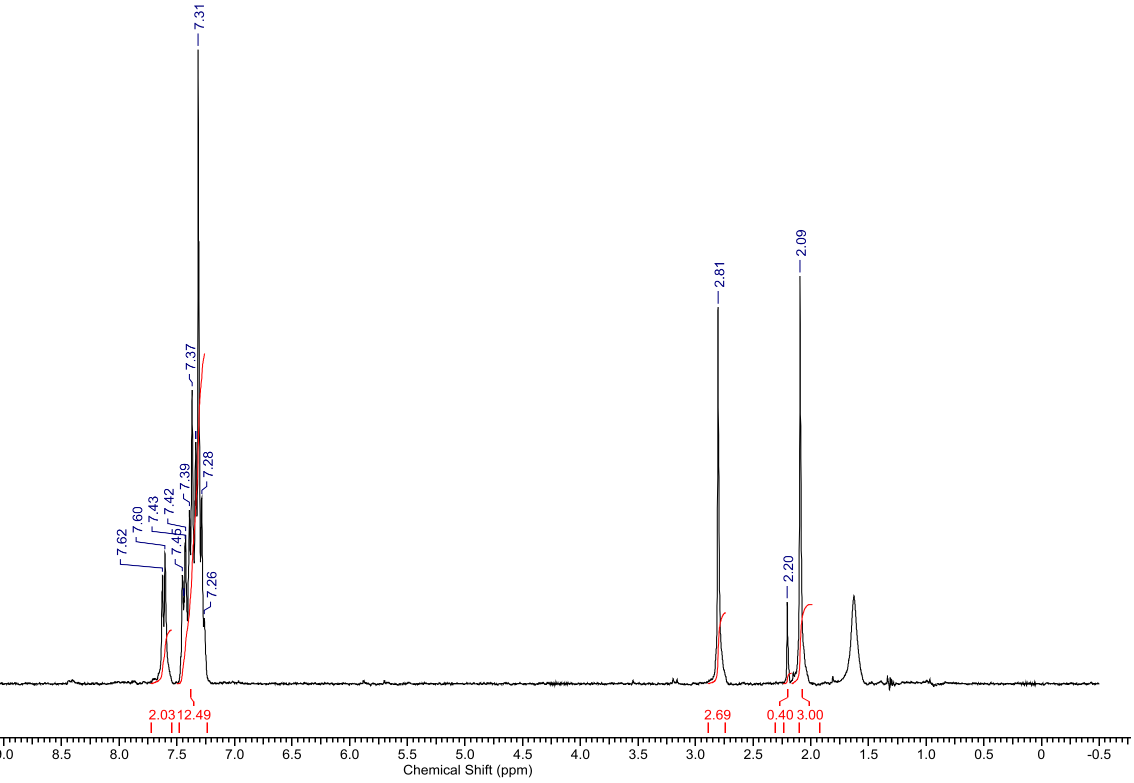

^{13}C NMR of **3ac**

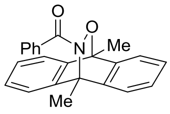

3ac

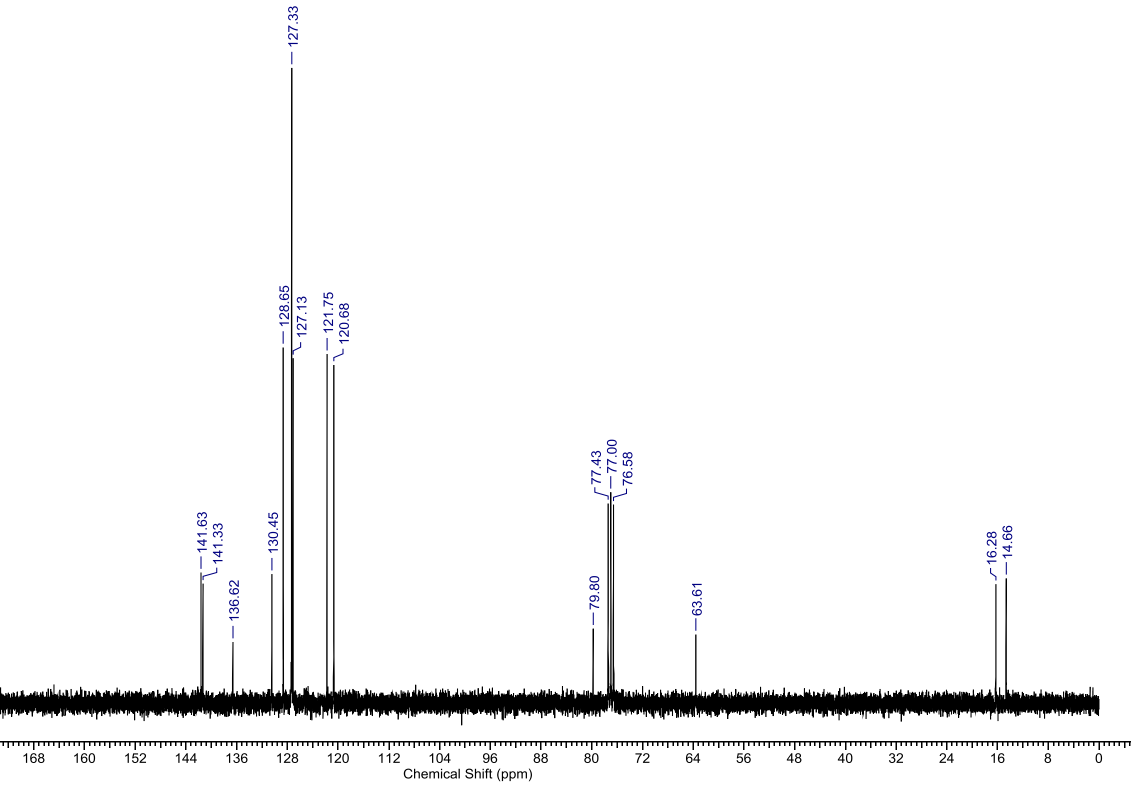

^{1}H NMR of **3bc**

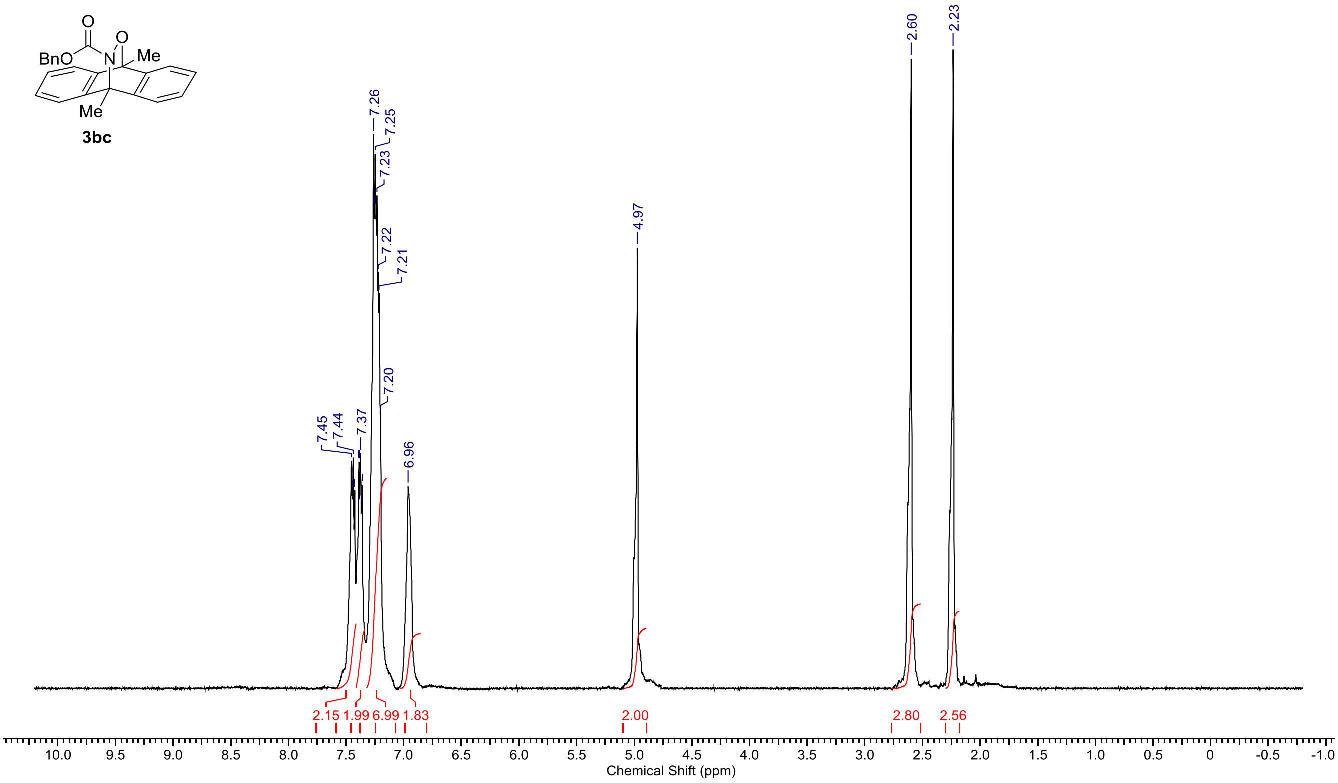

^{13}C NMR of **3bc**

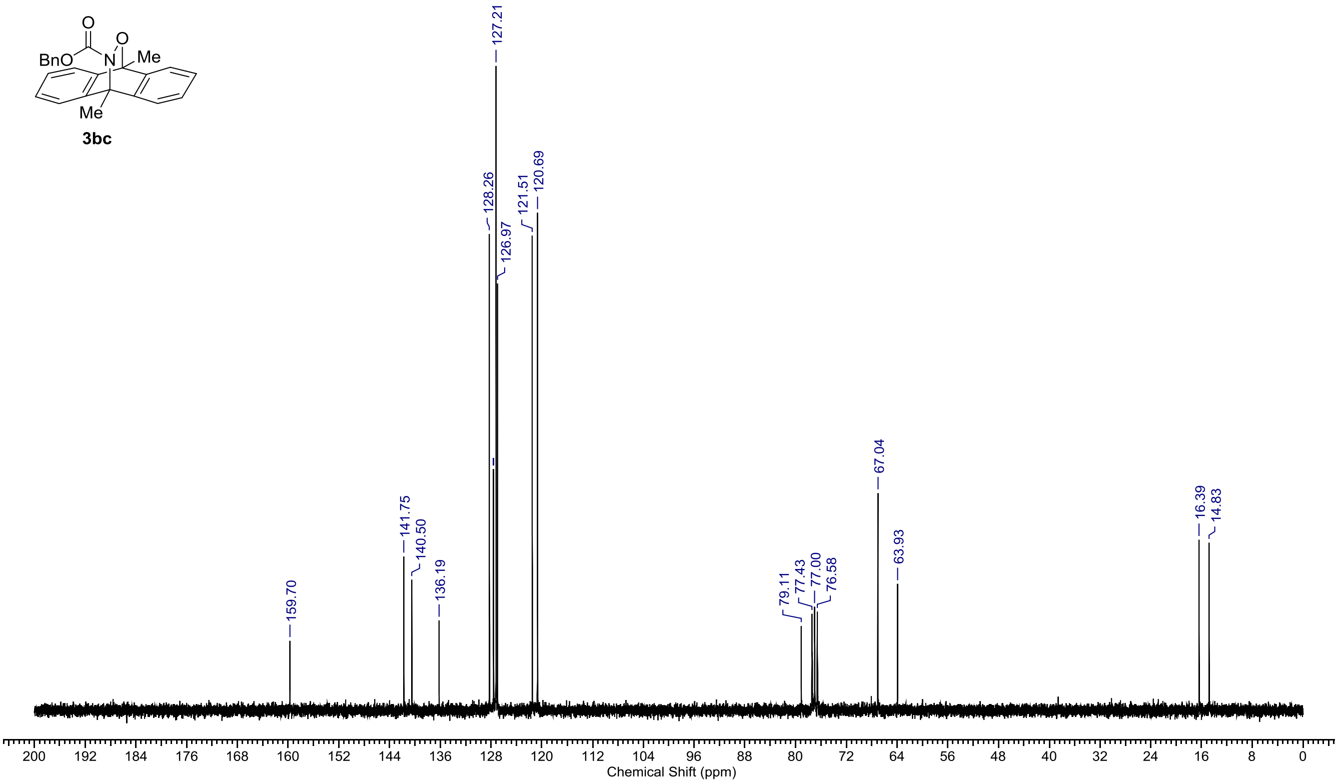

^{1}H NMR of **3cc**

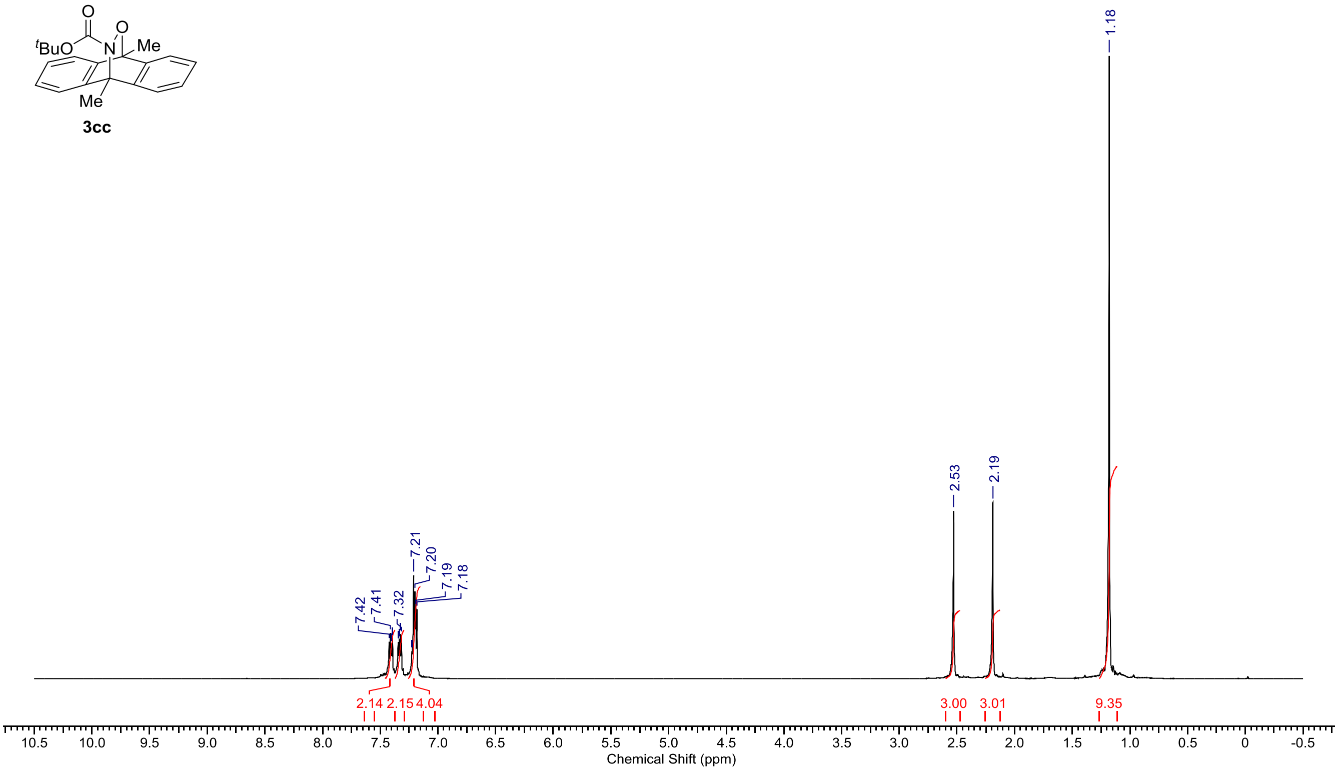

^{13}C NMR of **3cc**

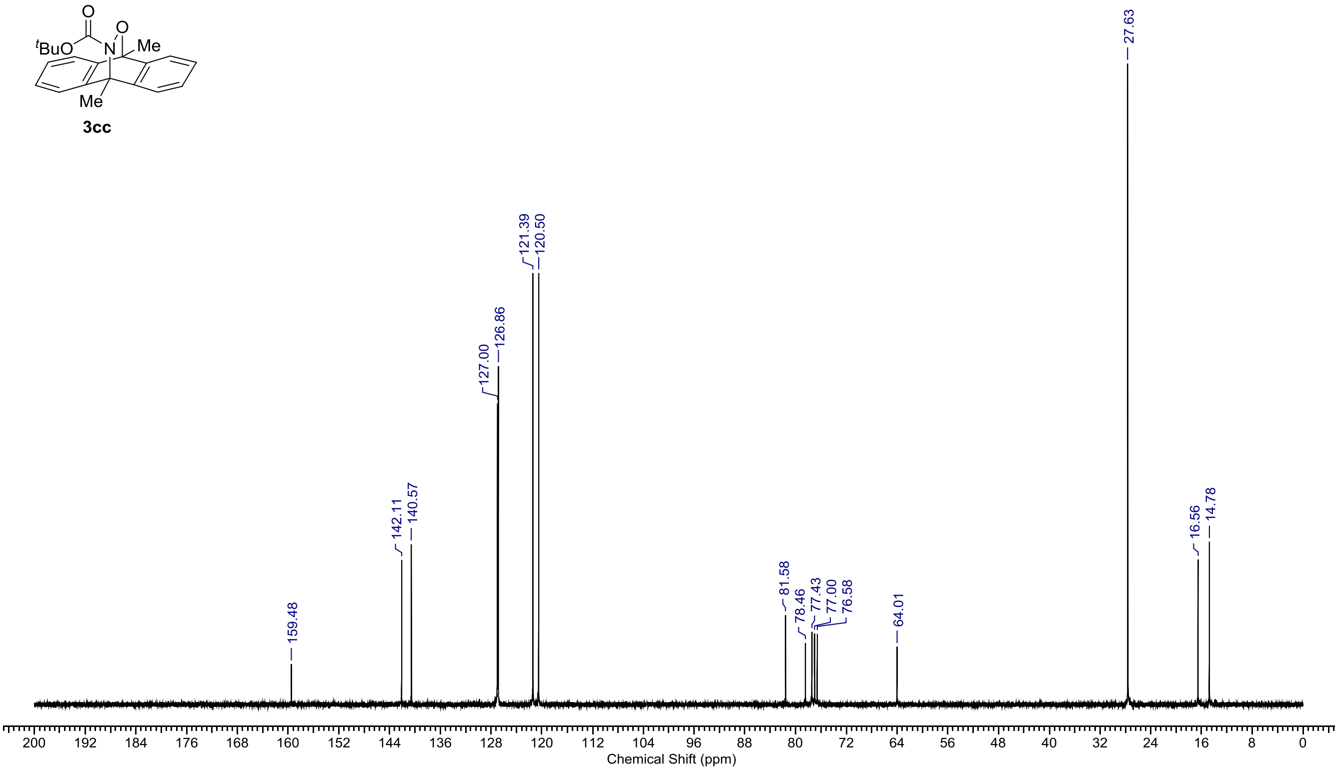

^{1}H NMR of **3ad**

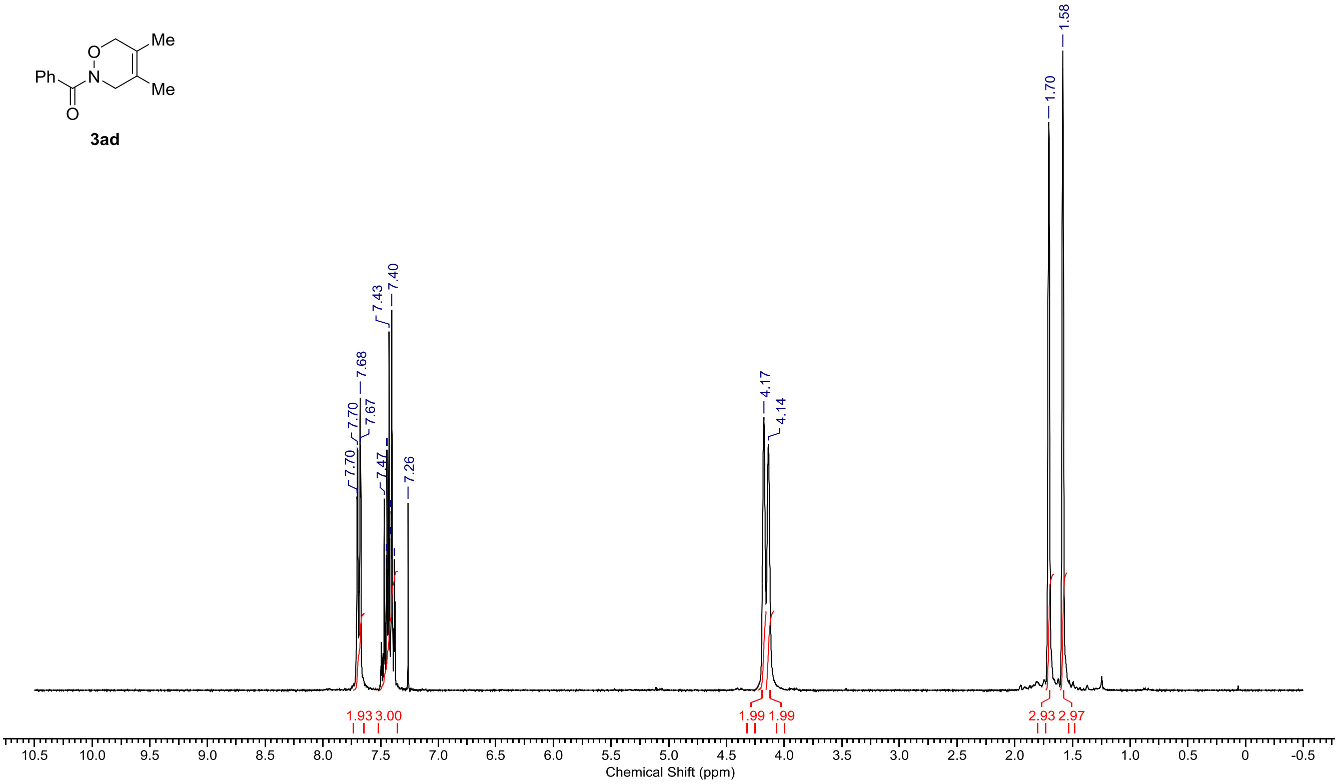

^{13}C NMR of **3ad**

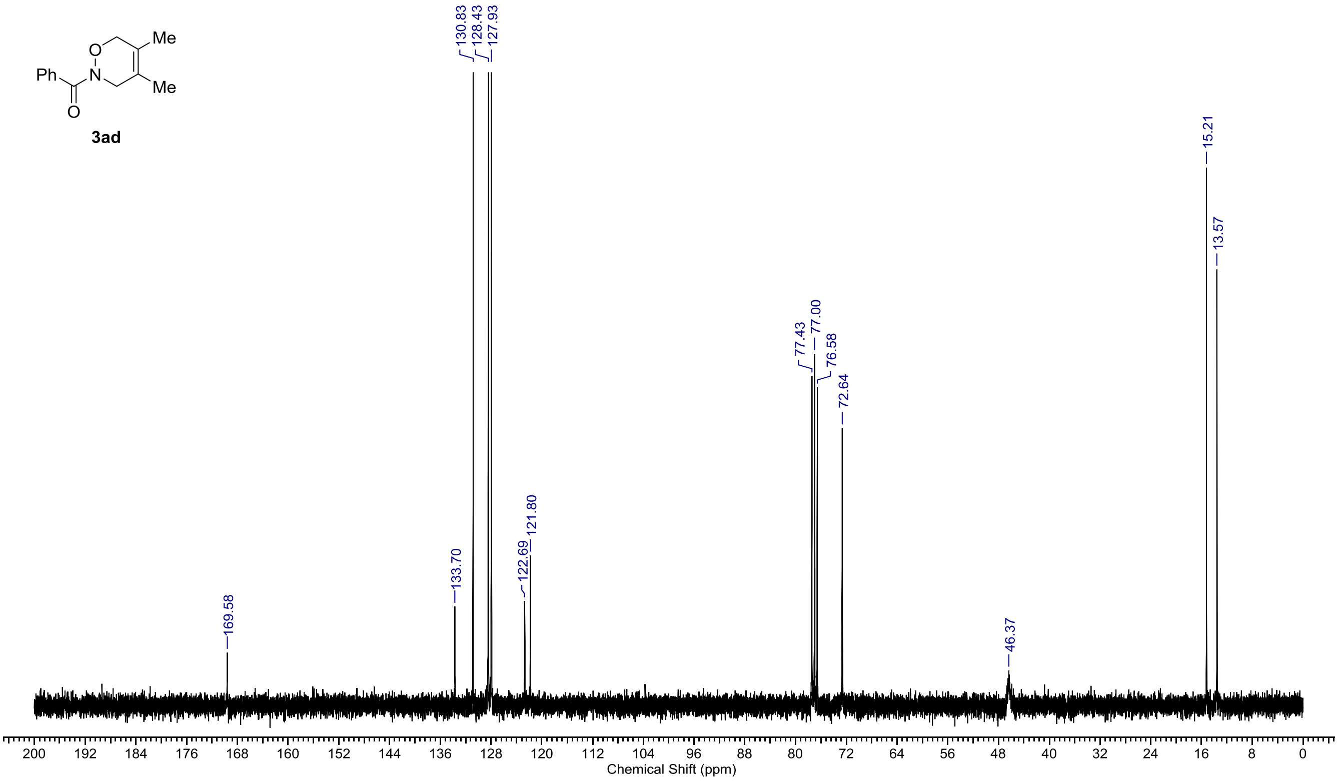

^{1}H NMR of **3bd**

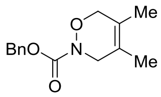

3bd

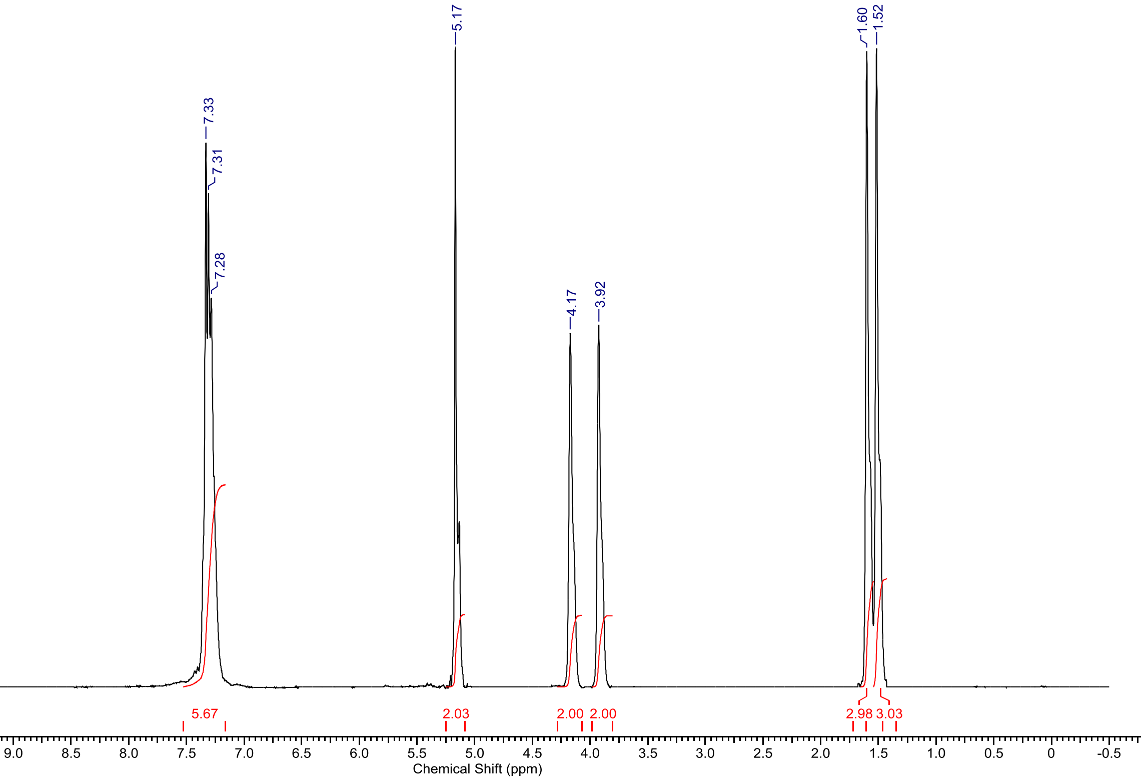

^{13}C NMR of **3bd**

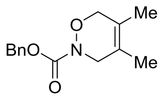

3bd

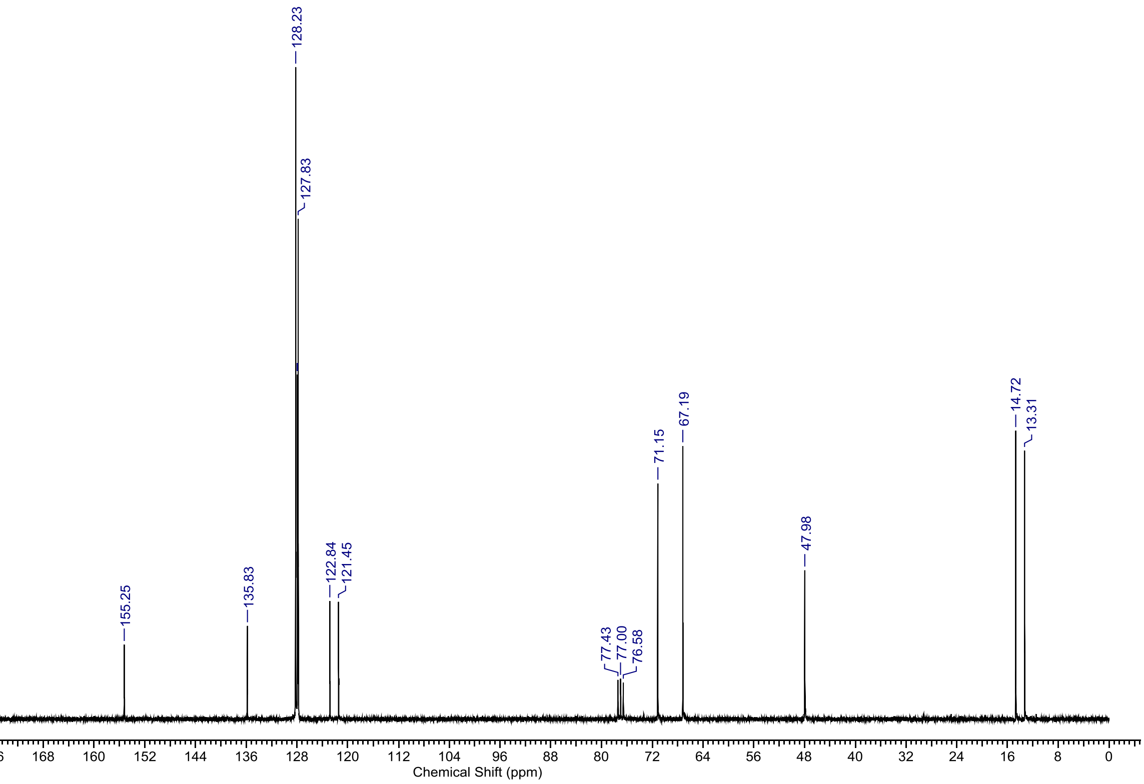

^{1}H NMR of **3cd**

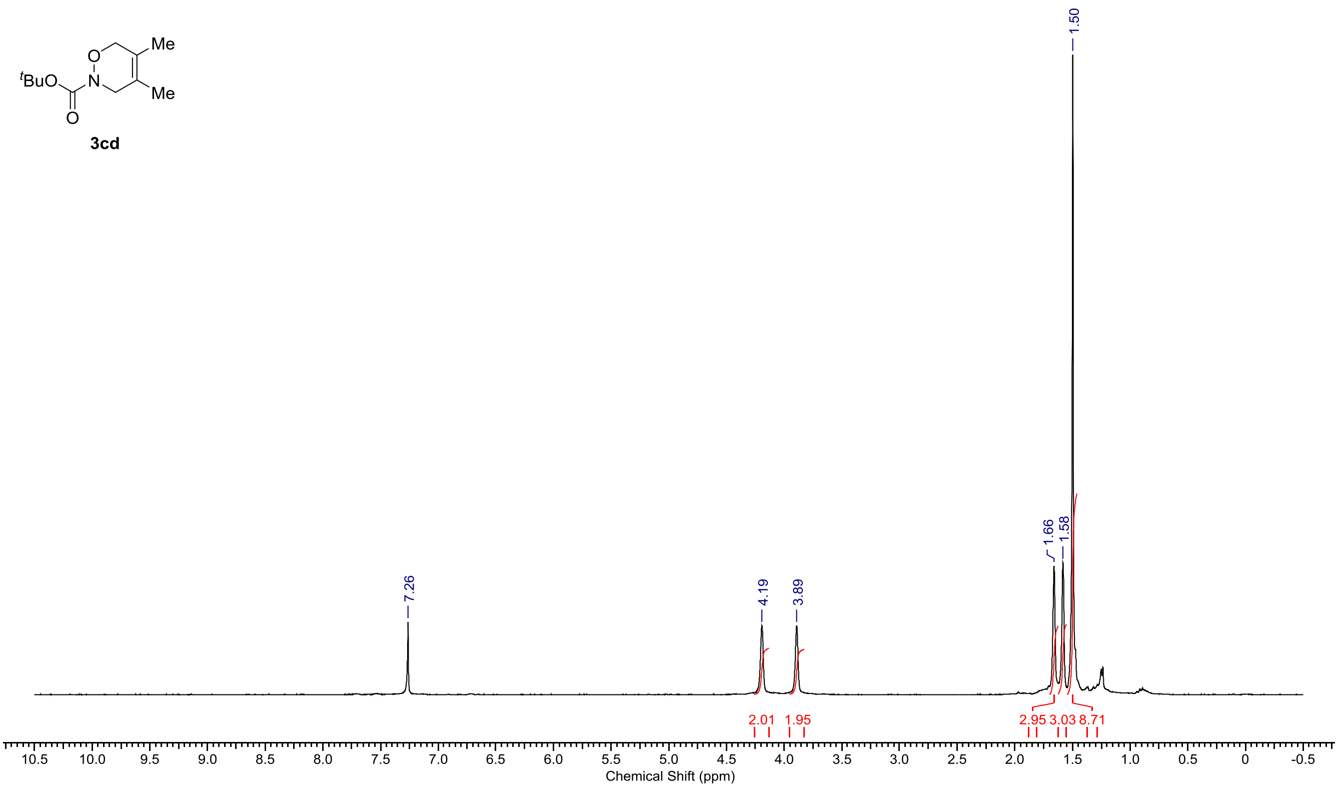

^{13}C NMR of **3cd**

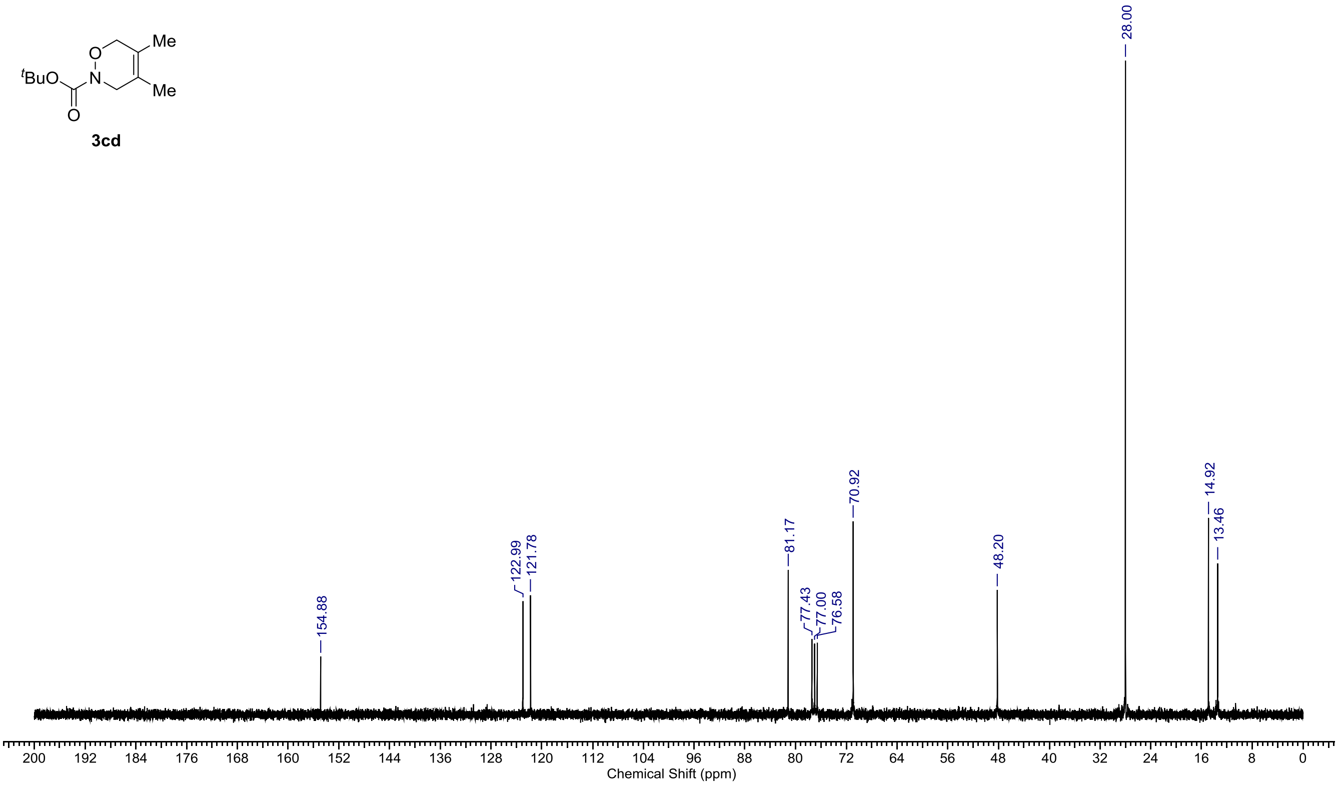

^{1}H NMR of **3ae** (regioisomeric mixture)

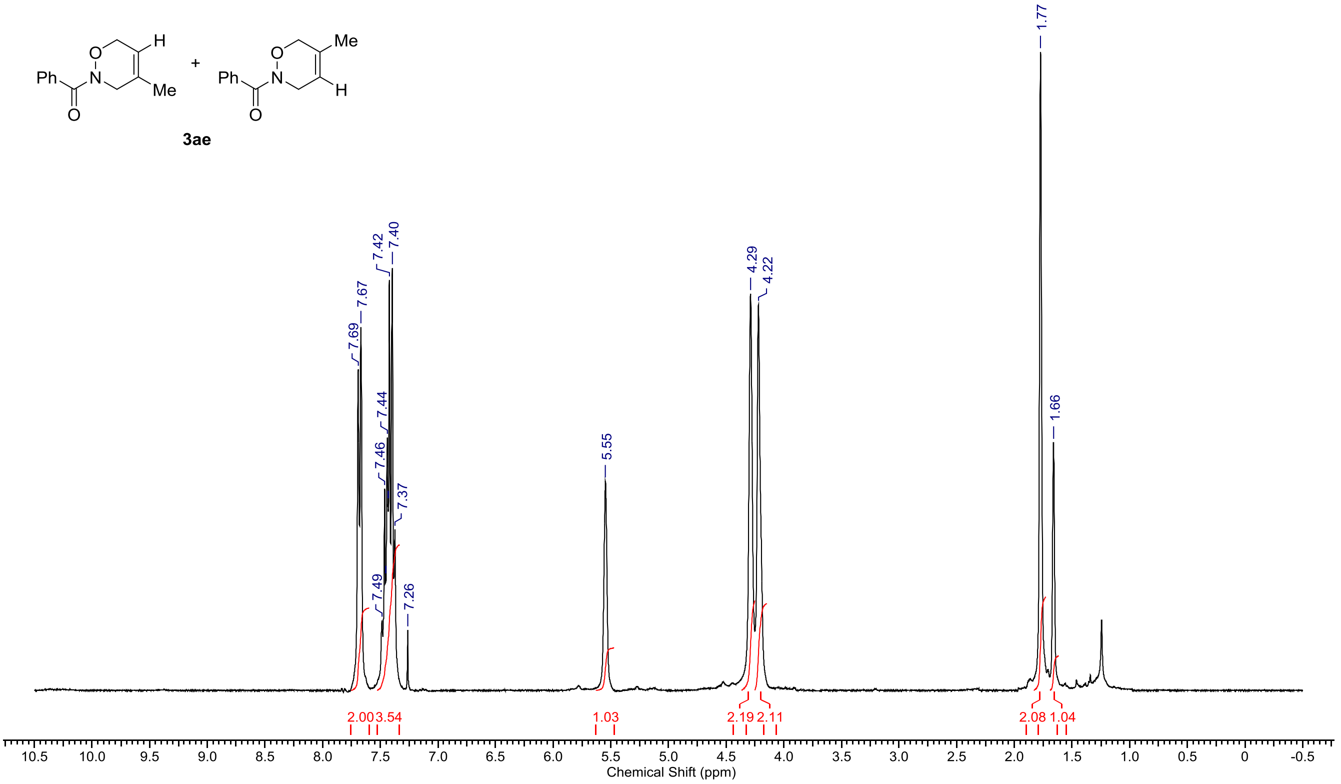

^{13}C NMR of **3ae** (regioisomeric mixture)

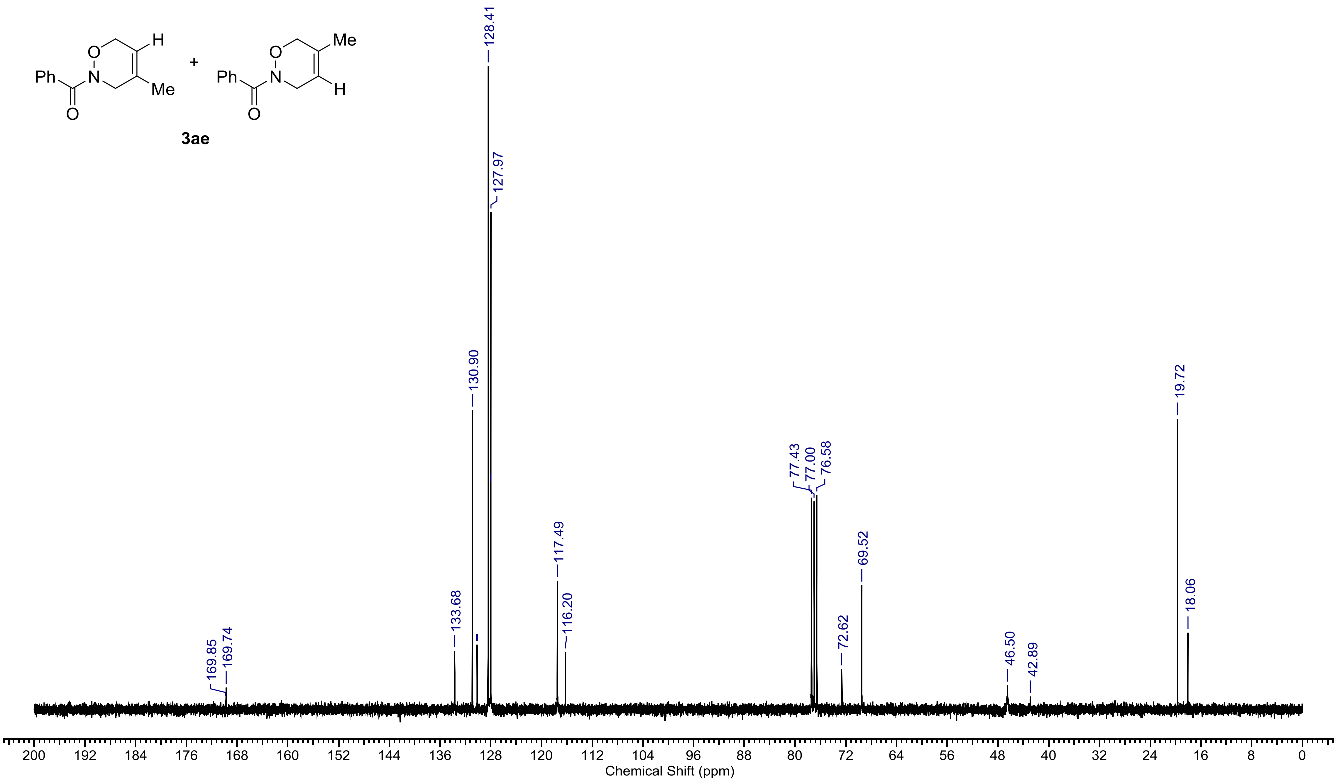

^{1}H NMR of **3be** (regioisomeric mixture)

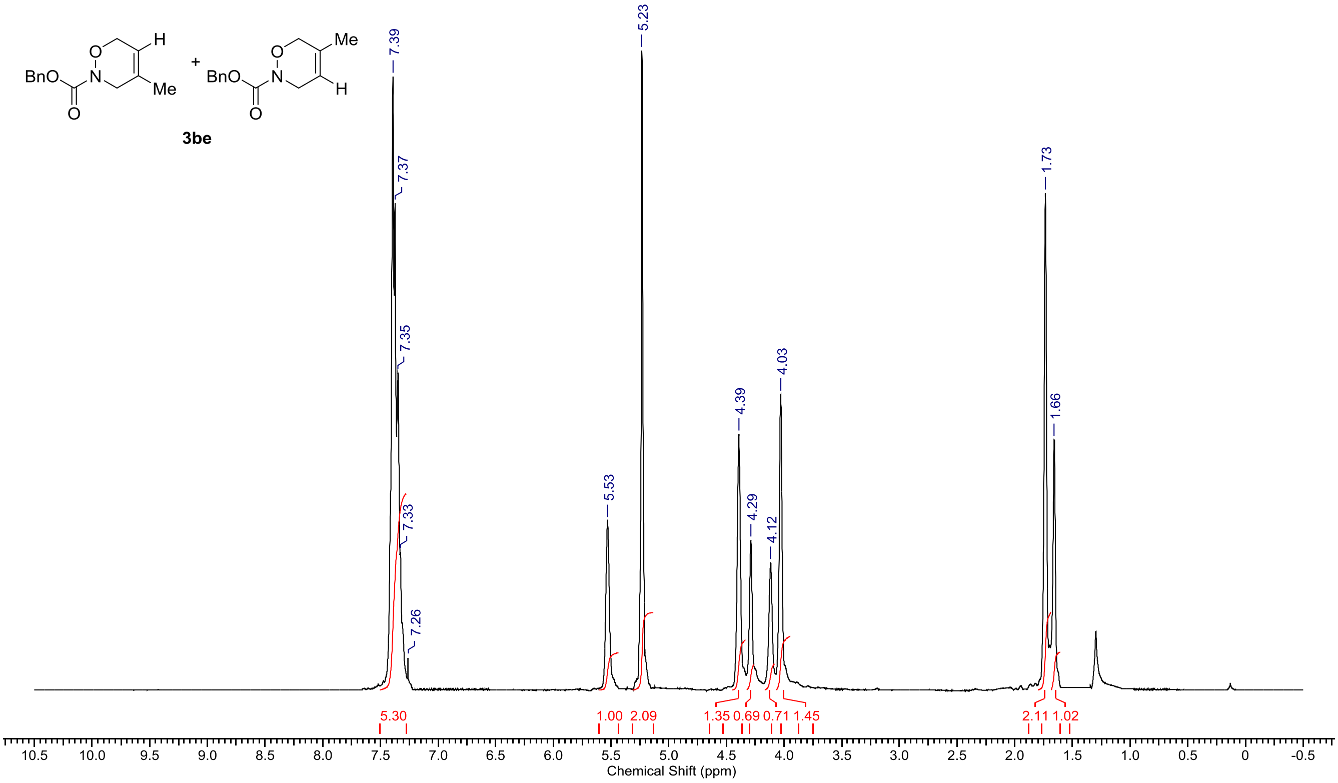

^{13}C NMR of **3be** (regioisomeric mixture)

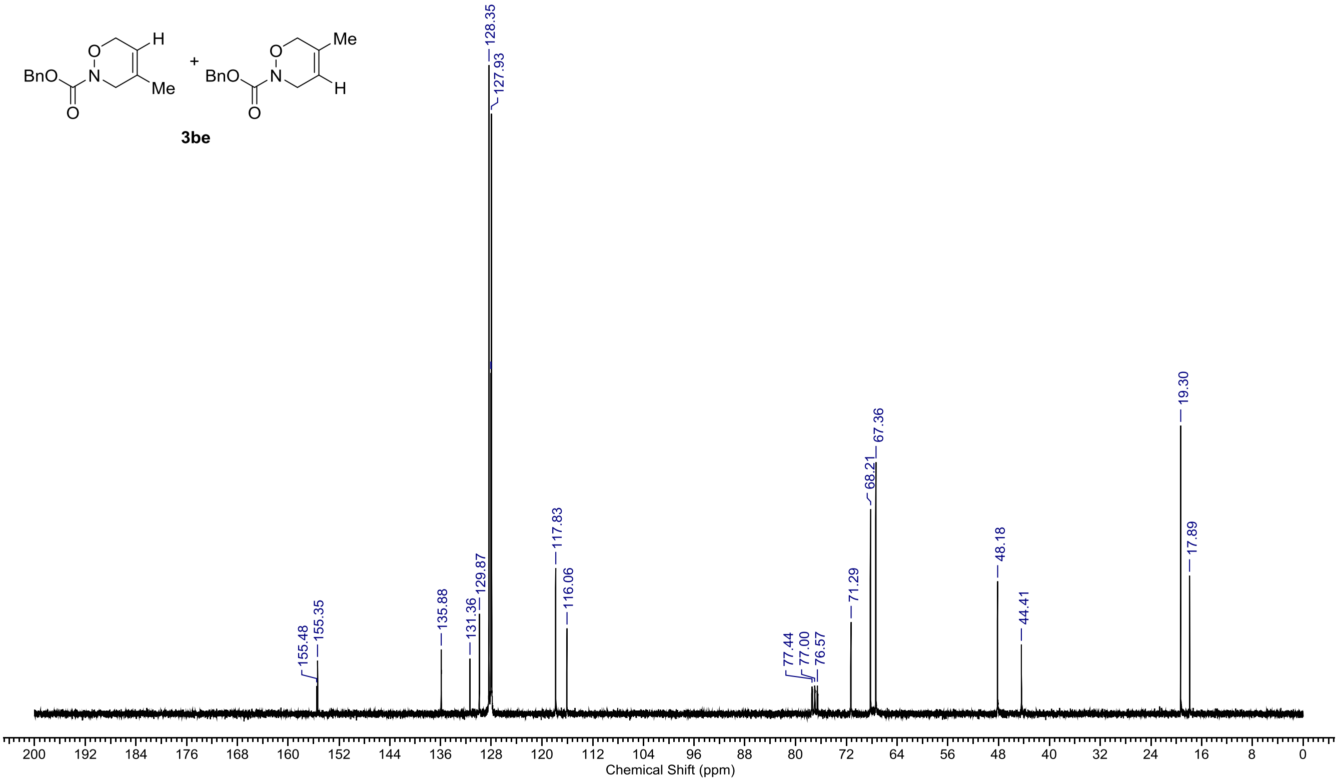

^{1}H NMR of **3ce** (regioisomeric mixture)

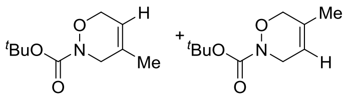

3ce

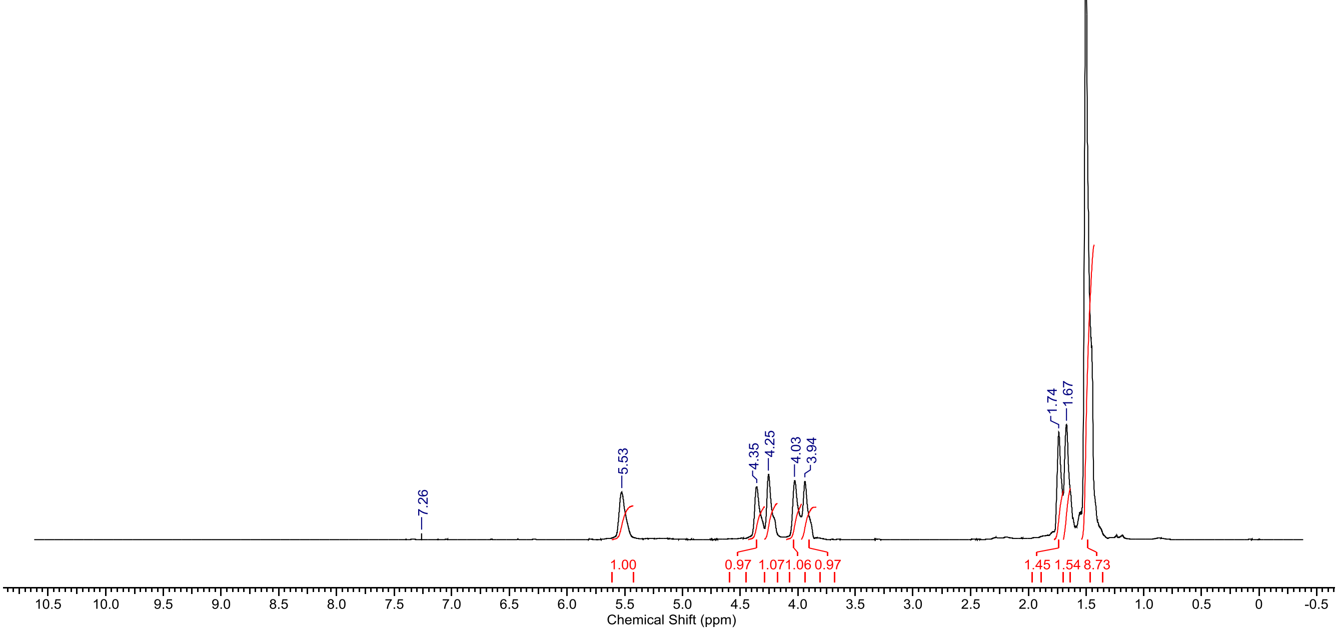

^{13}C NMR of **3ce** (regioisomeric mixture)

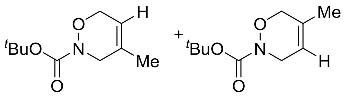

3ce

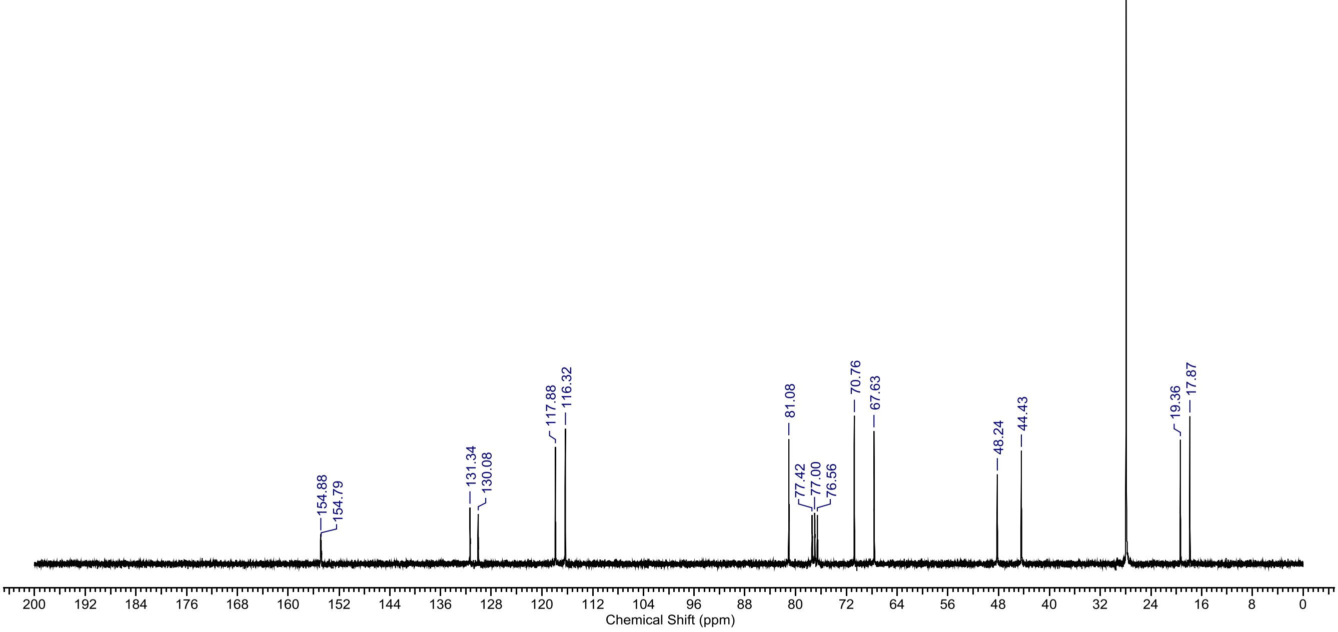

^{1}H NMR of **4**

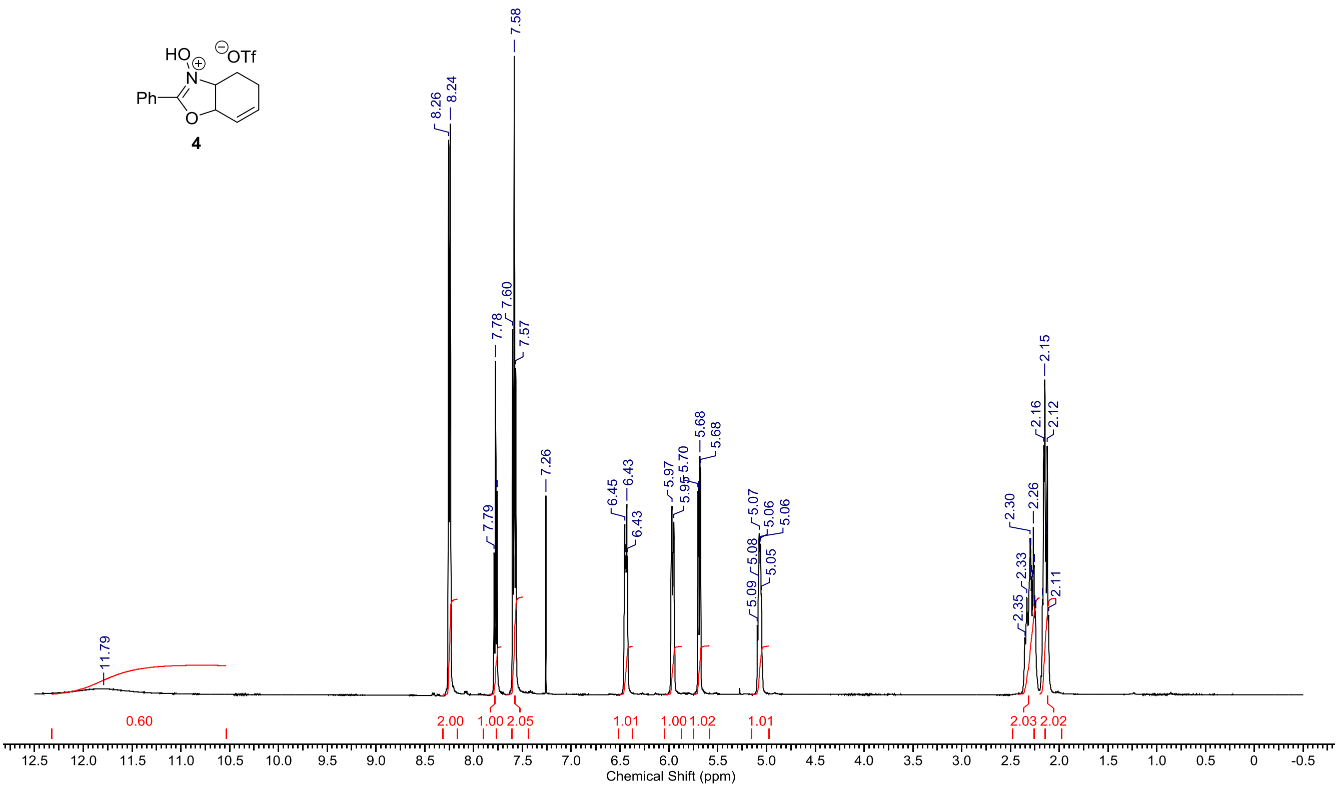

^{13}C NMR of **4**

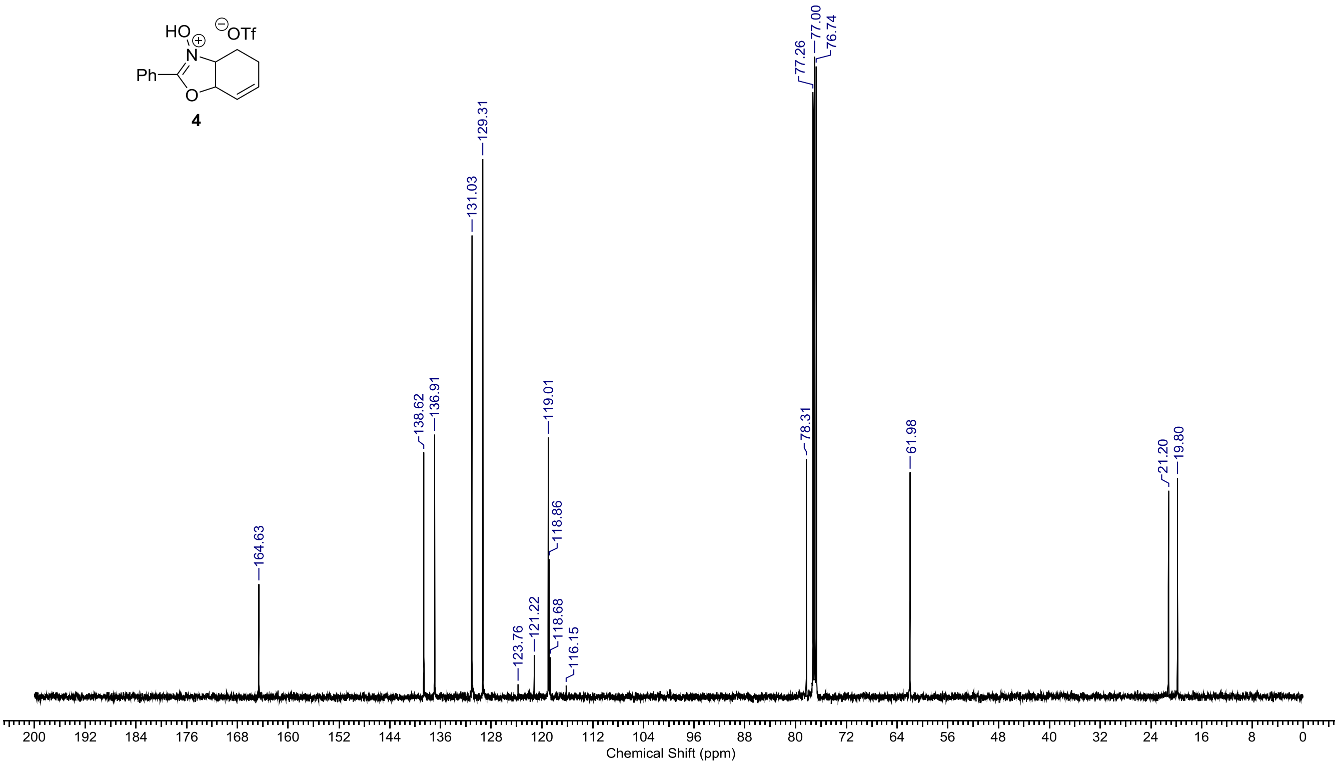

^{1}H NMR of **6aa**

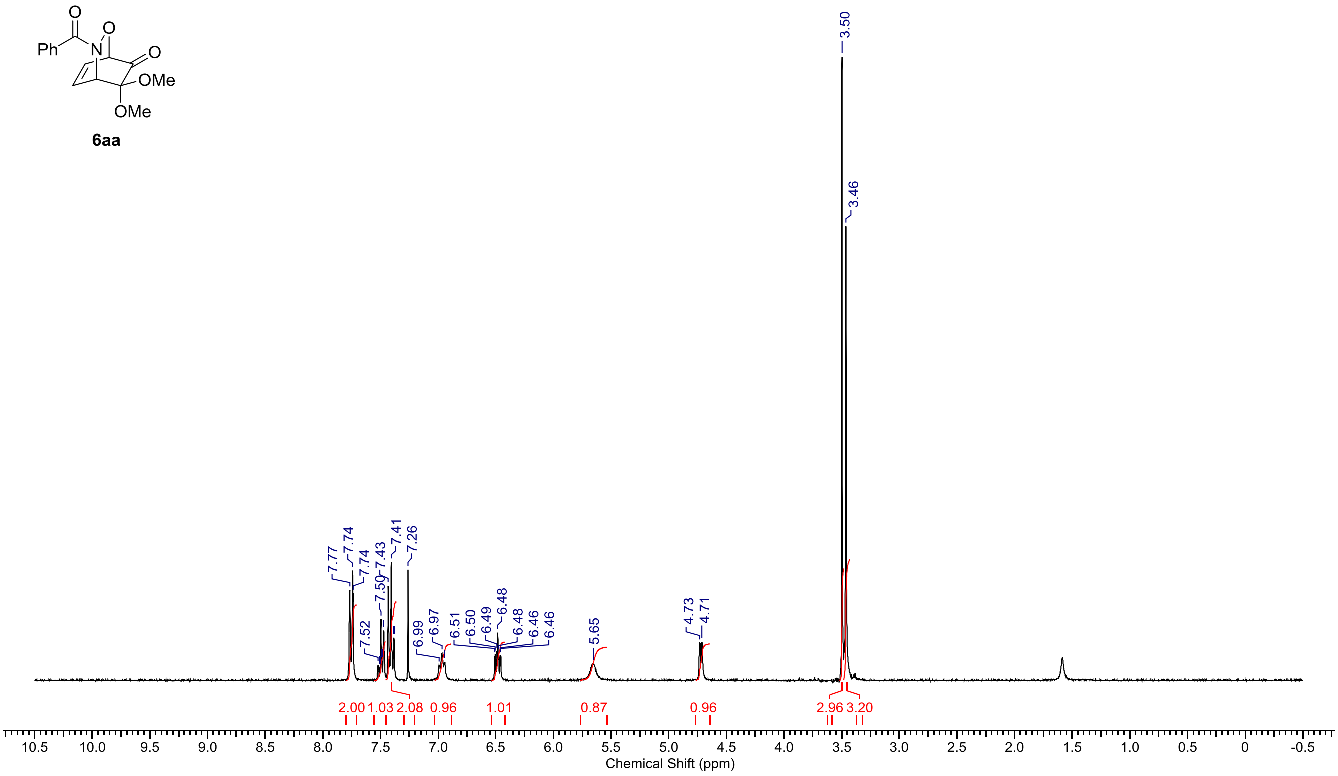

^{13}C NMR of **6aa**

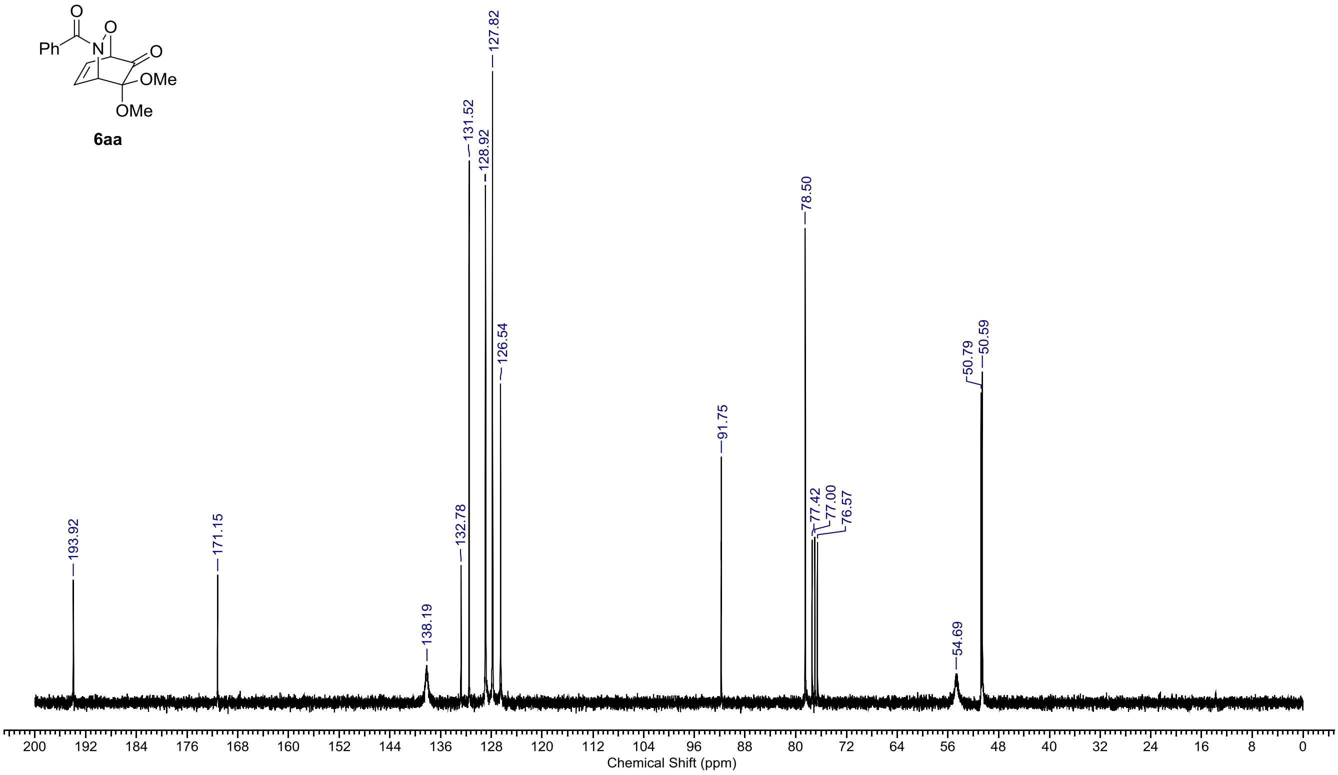

^{1}H NMR of **6ab**

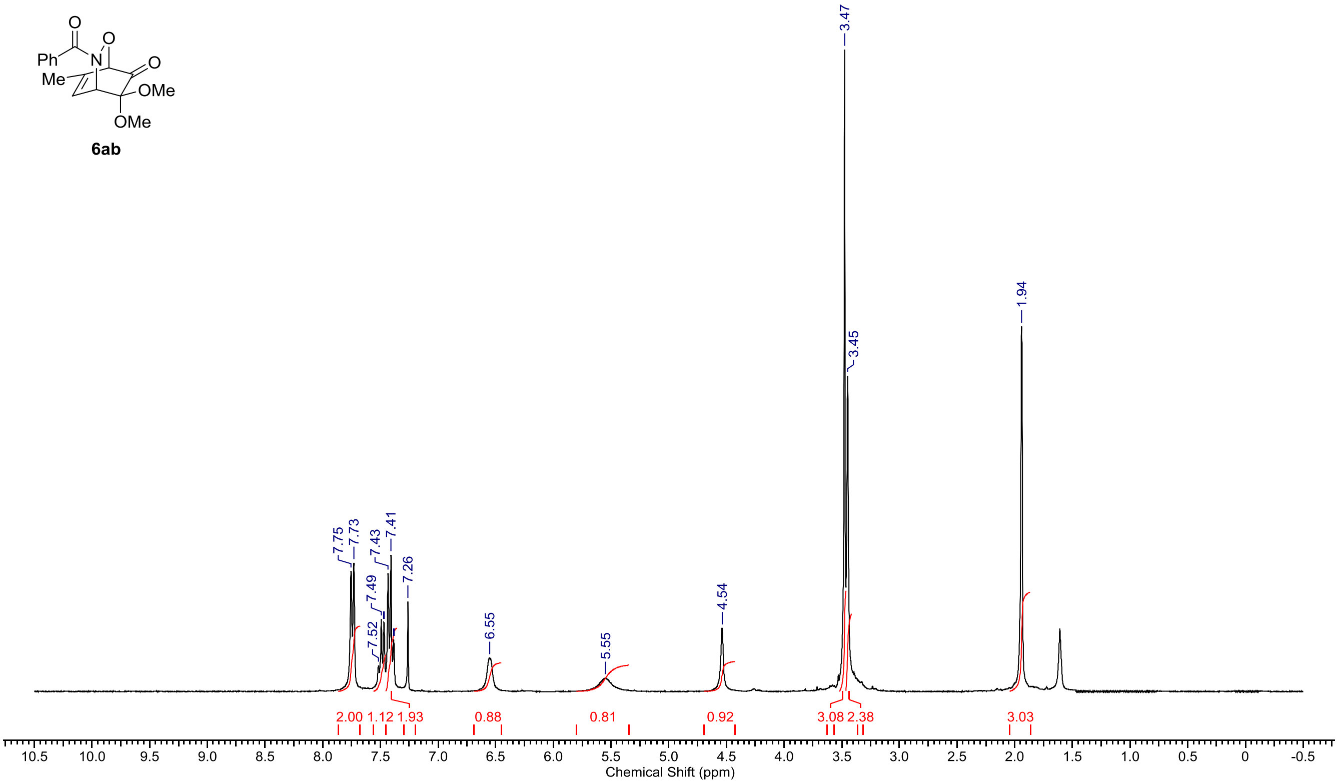

^{13}C NMR of **6ab**

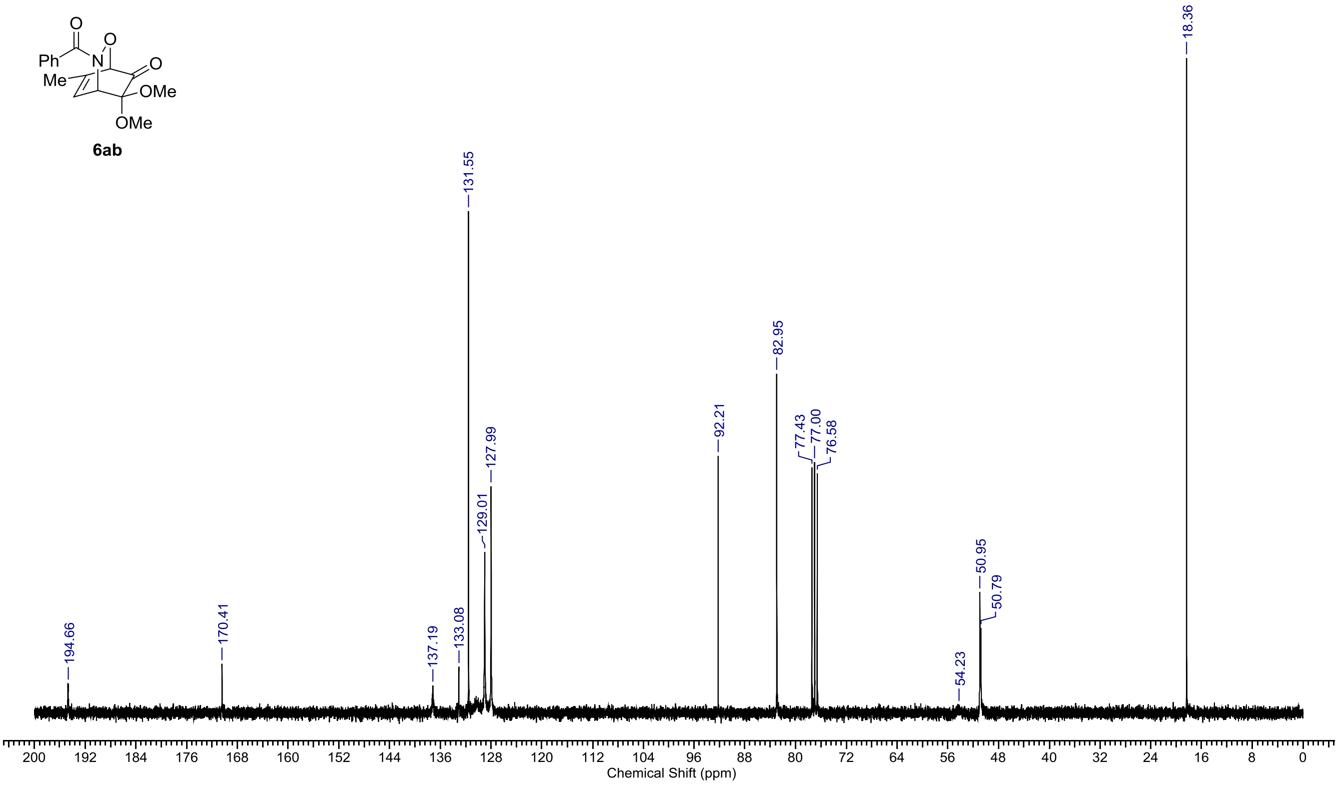

^{1}H NMR of **6ac**

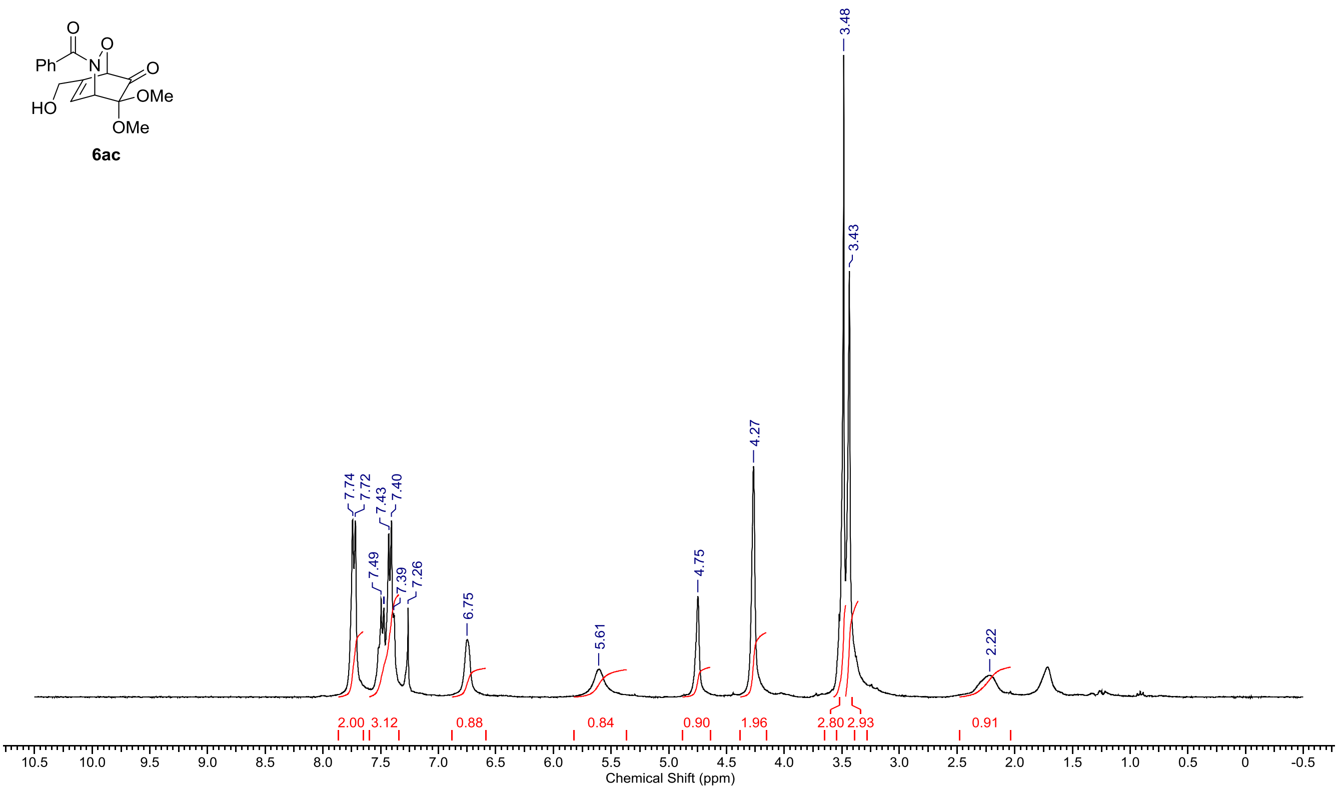

^{13}C NMR of **6ac**

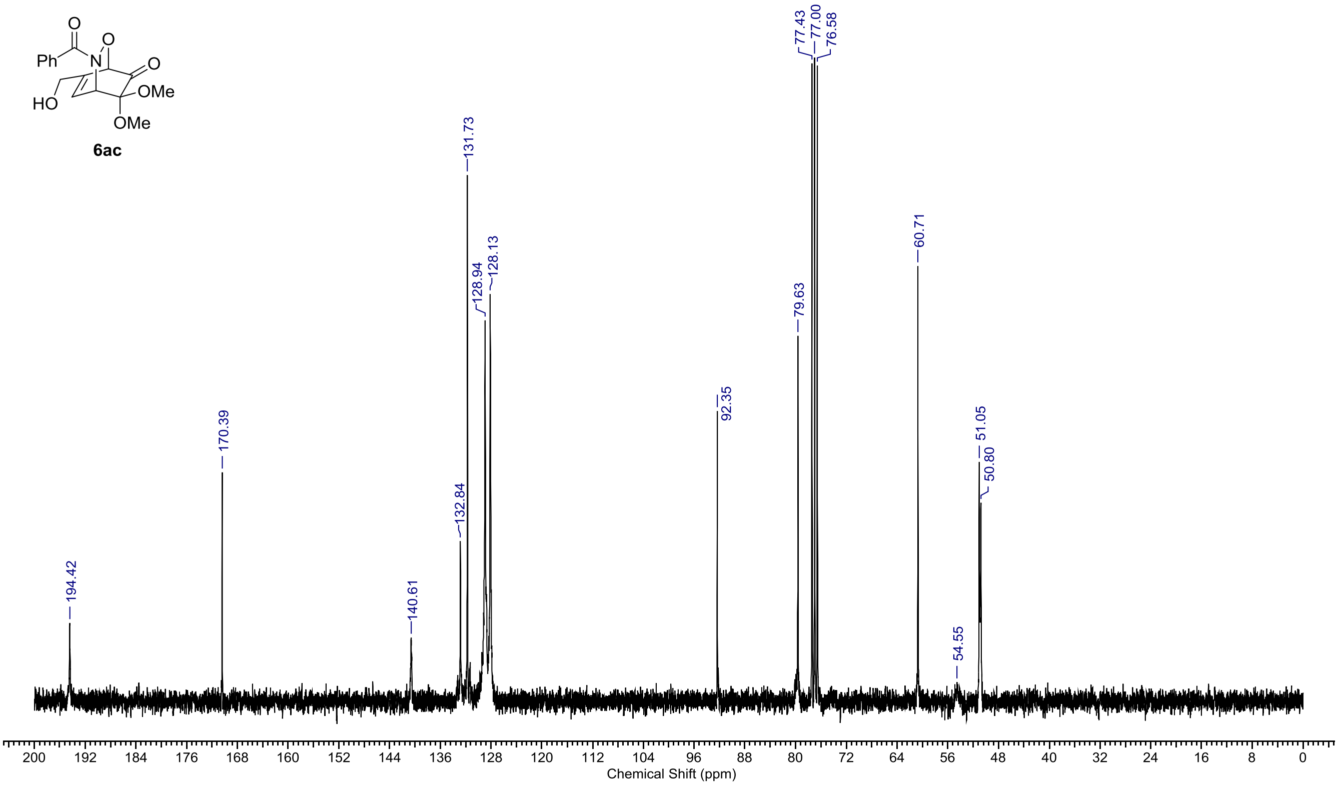

^{1}H NMR of **6ad**

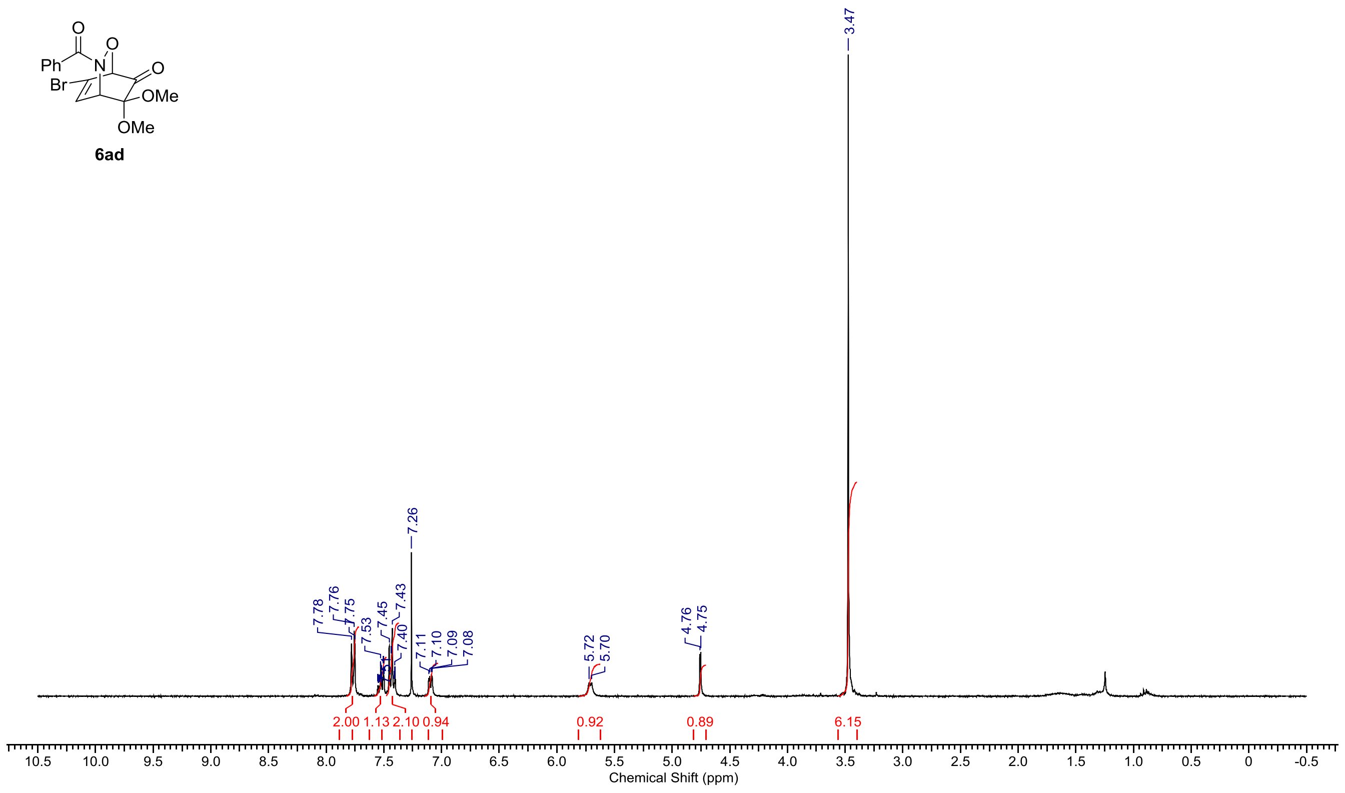

^{13}C NMR of **6ad**

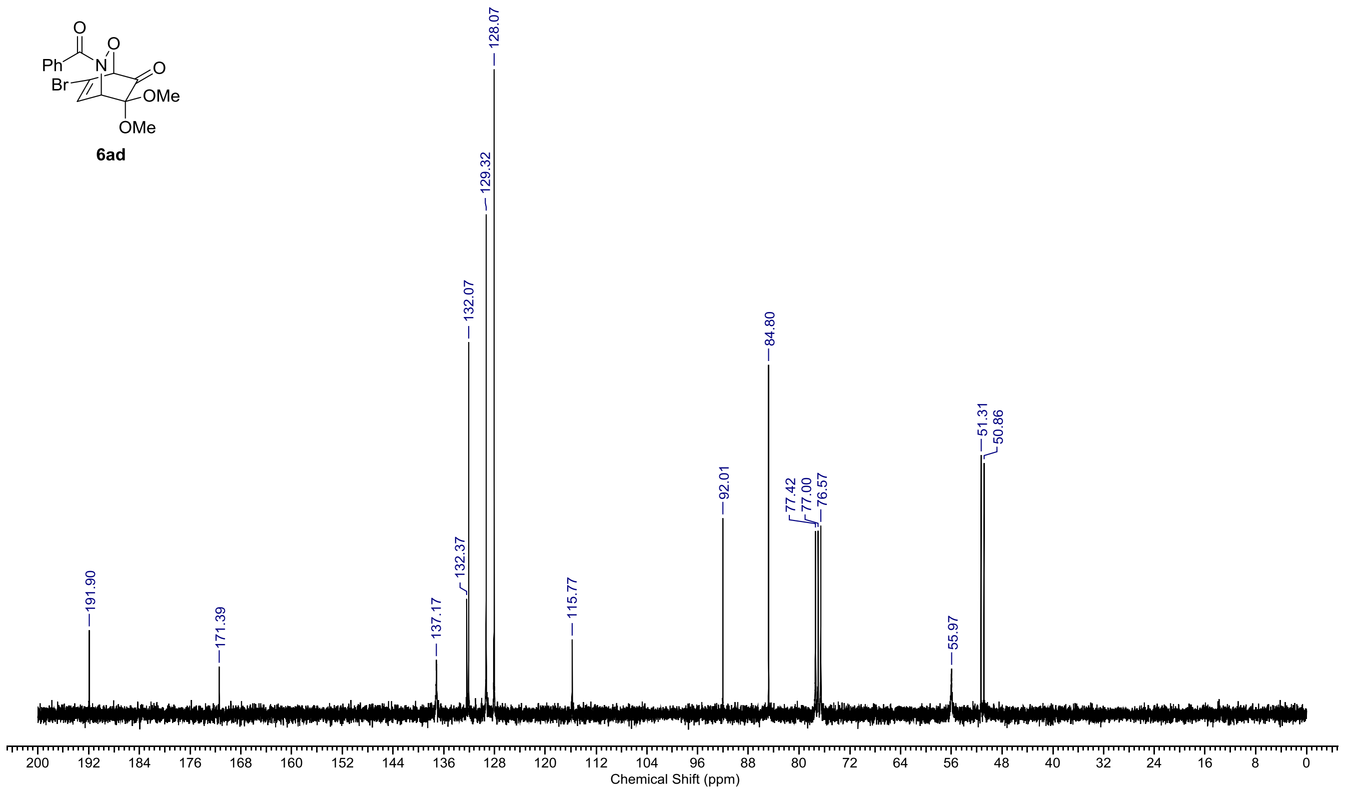

^{1}H NMR of **6ae**

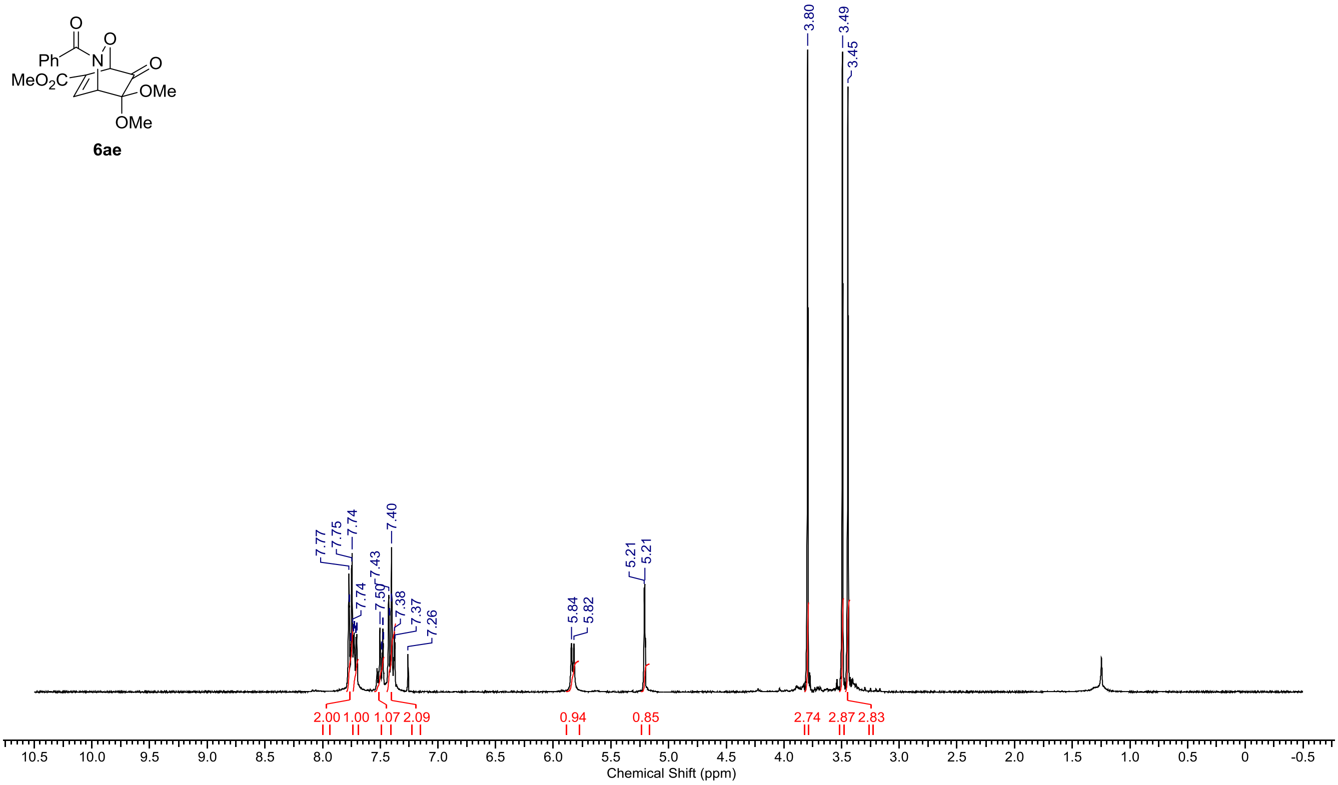

^{13}C NMR of **6ae**

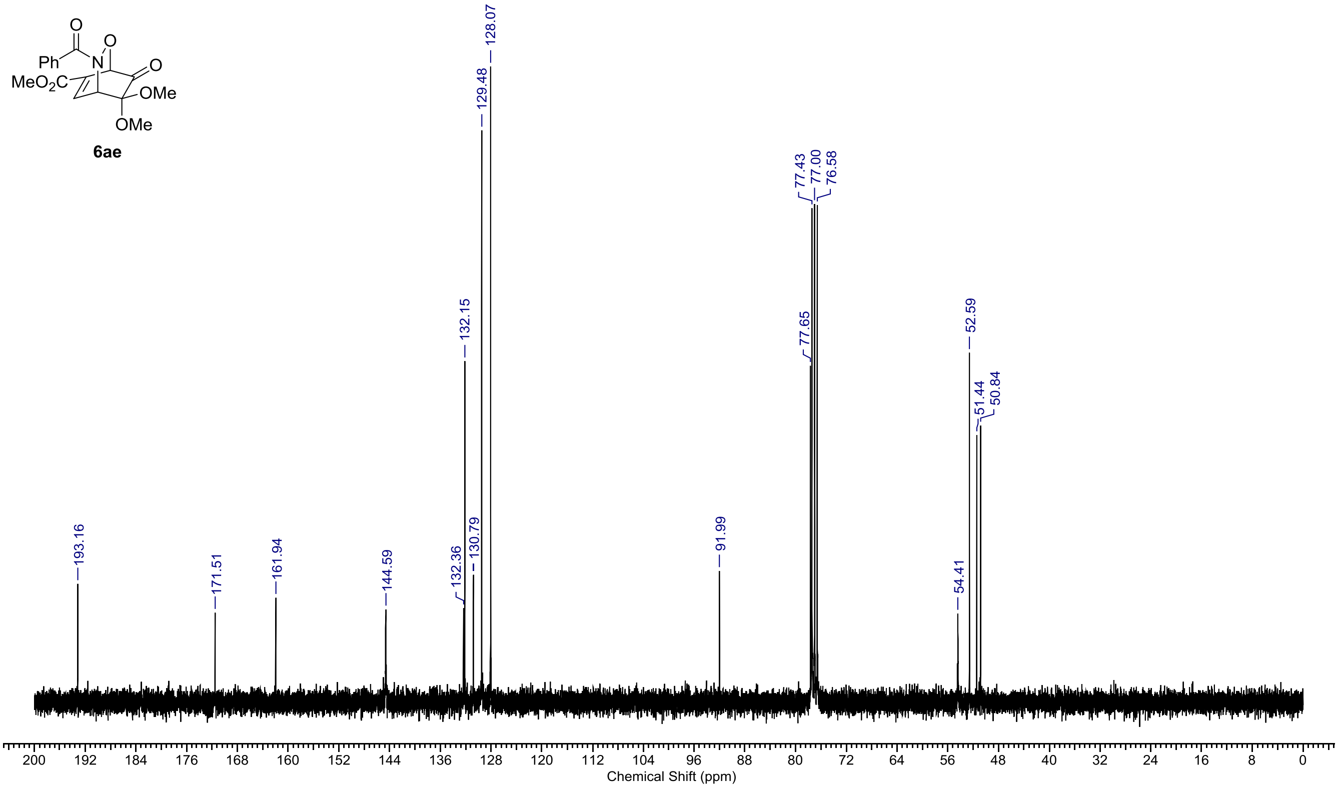

^{1}H NMR of **6af**

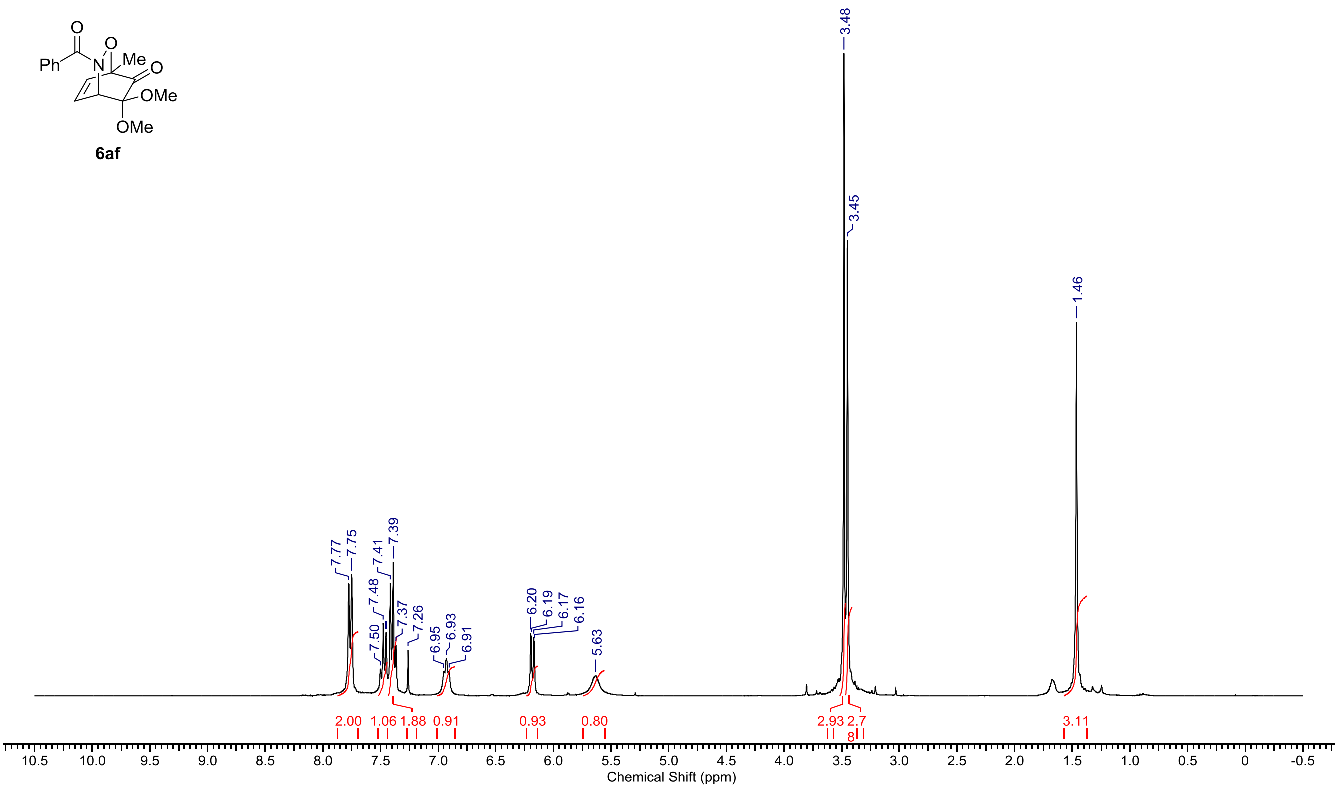

^{13}C NMR of **6af**

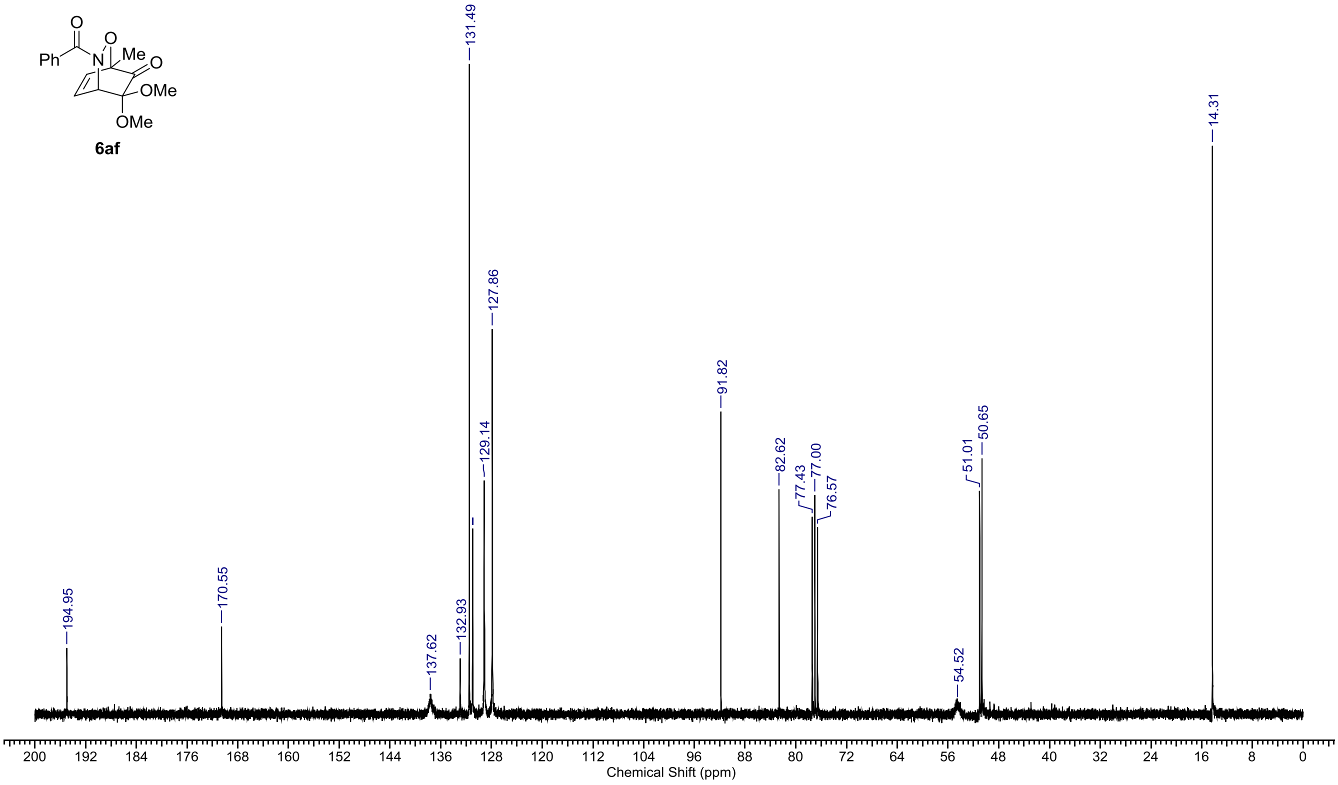

^{1}H NMR of **6ag**

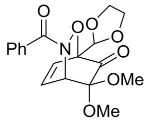

6ag

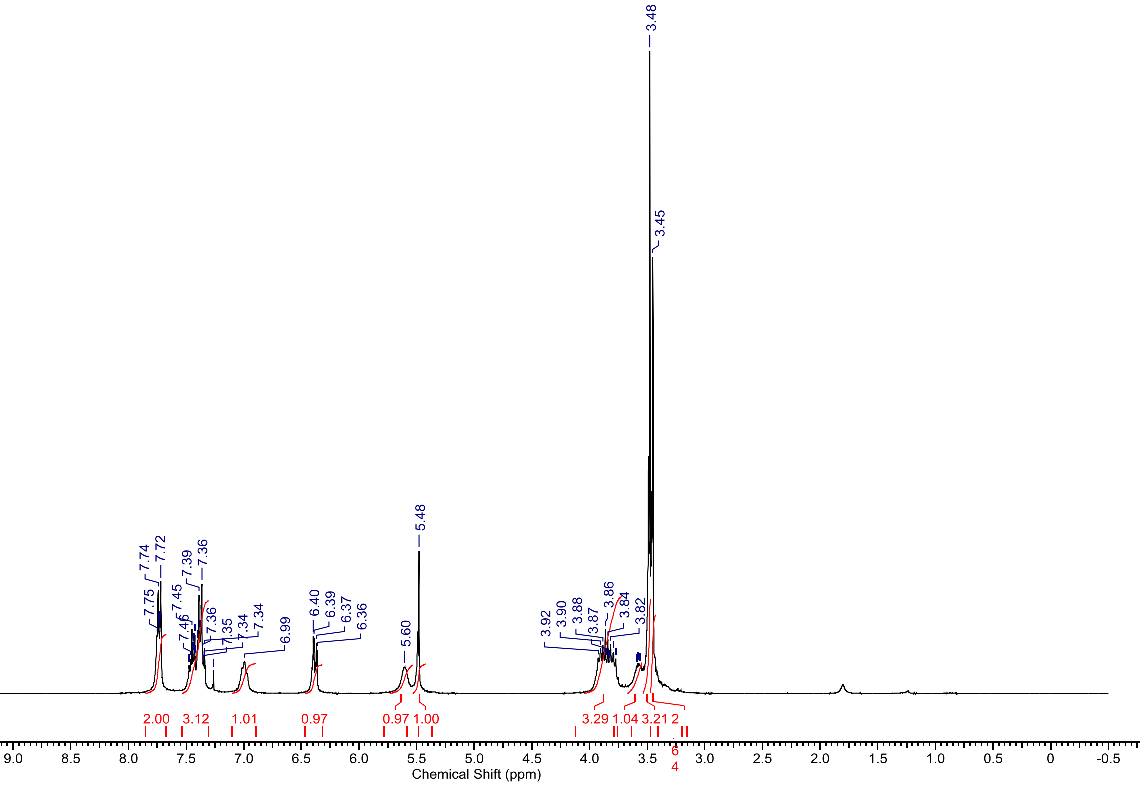

^{13}C NMR of **6ag**

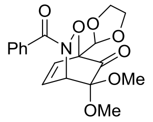

6ag

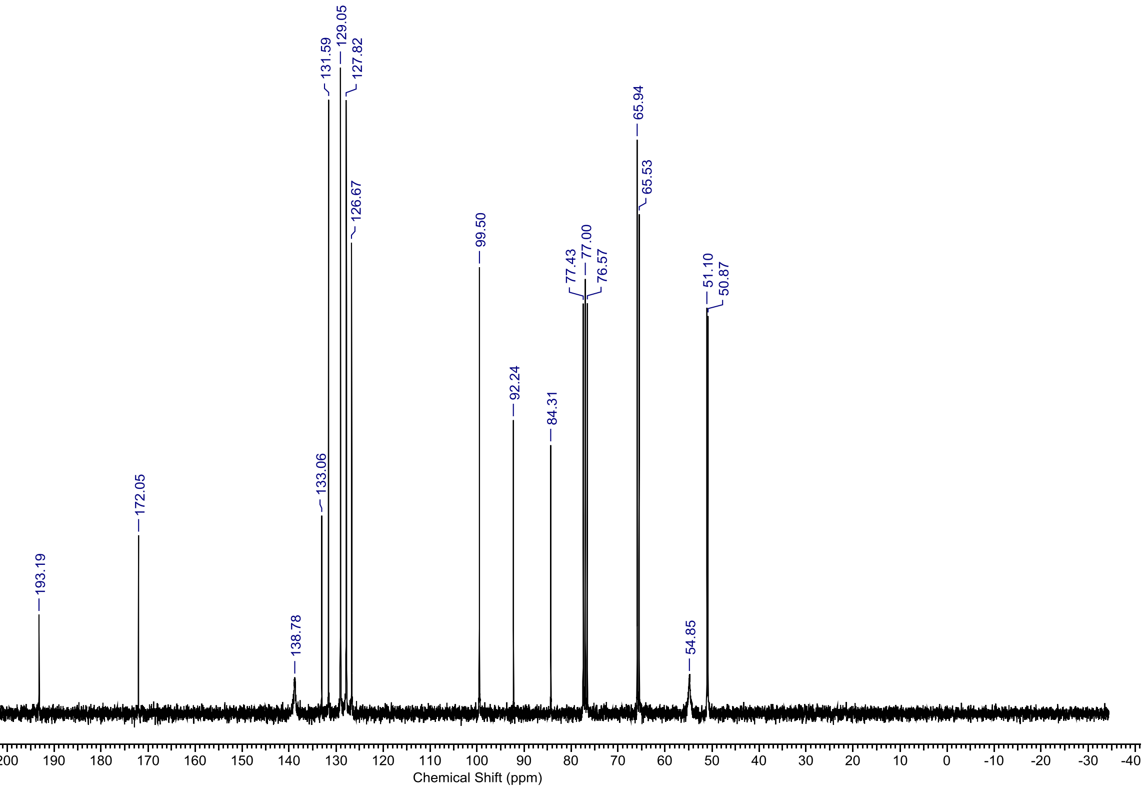

^{1}H NMR of **6ba**

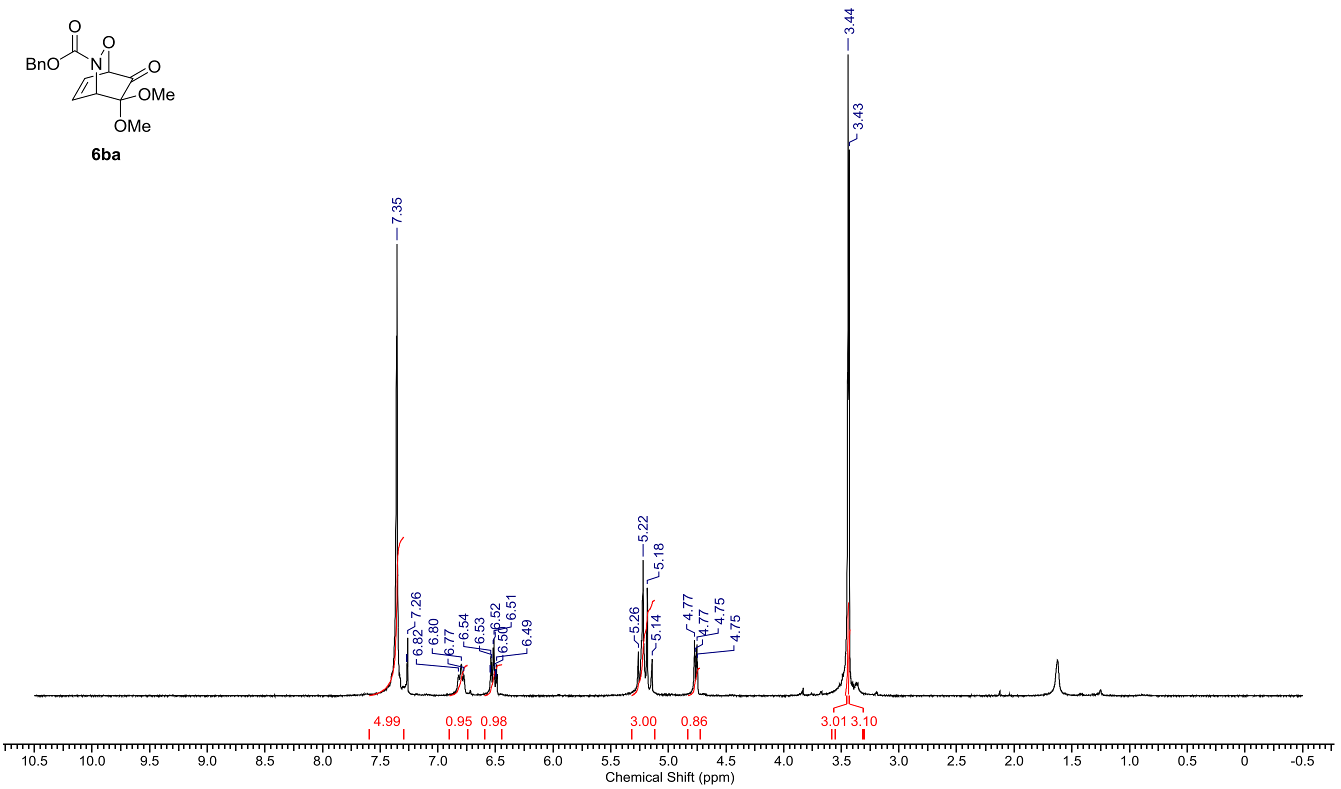

^{13}C NMR of **6ba**

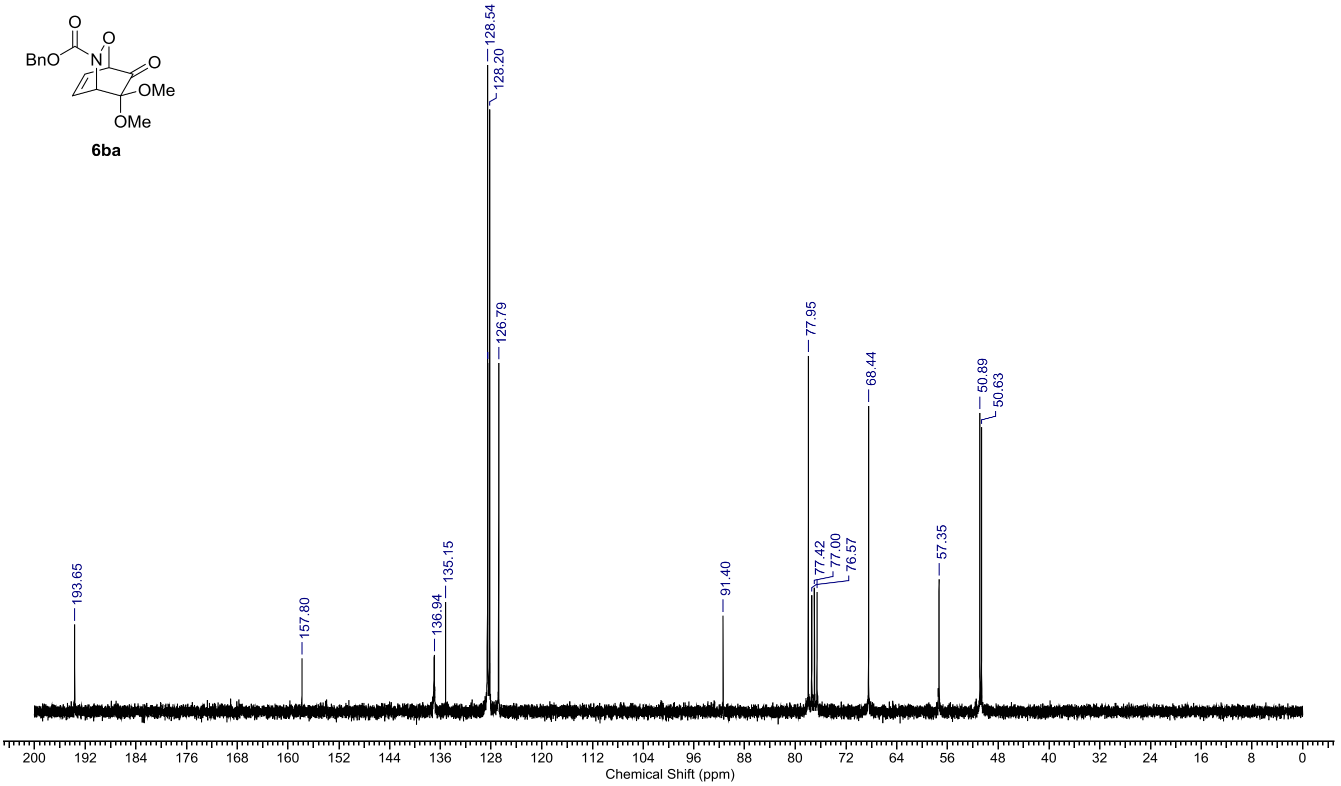

^{1}H NMR of **6ca**

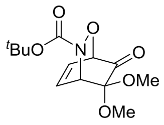

6ca

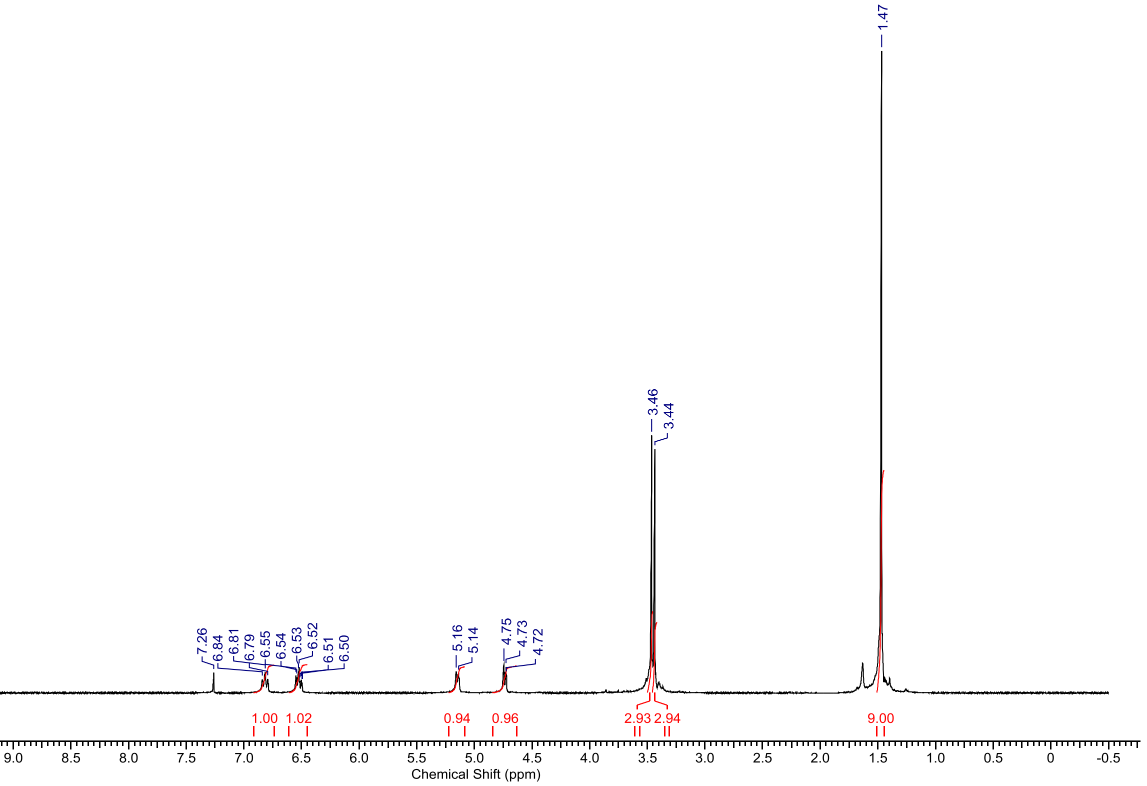

^{13}C NMR of **6ca**

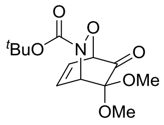

6ca

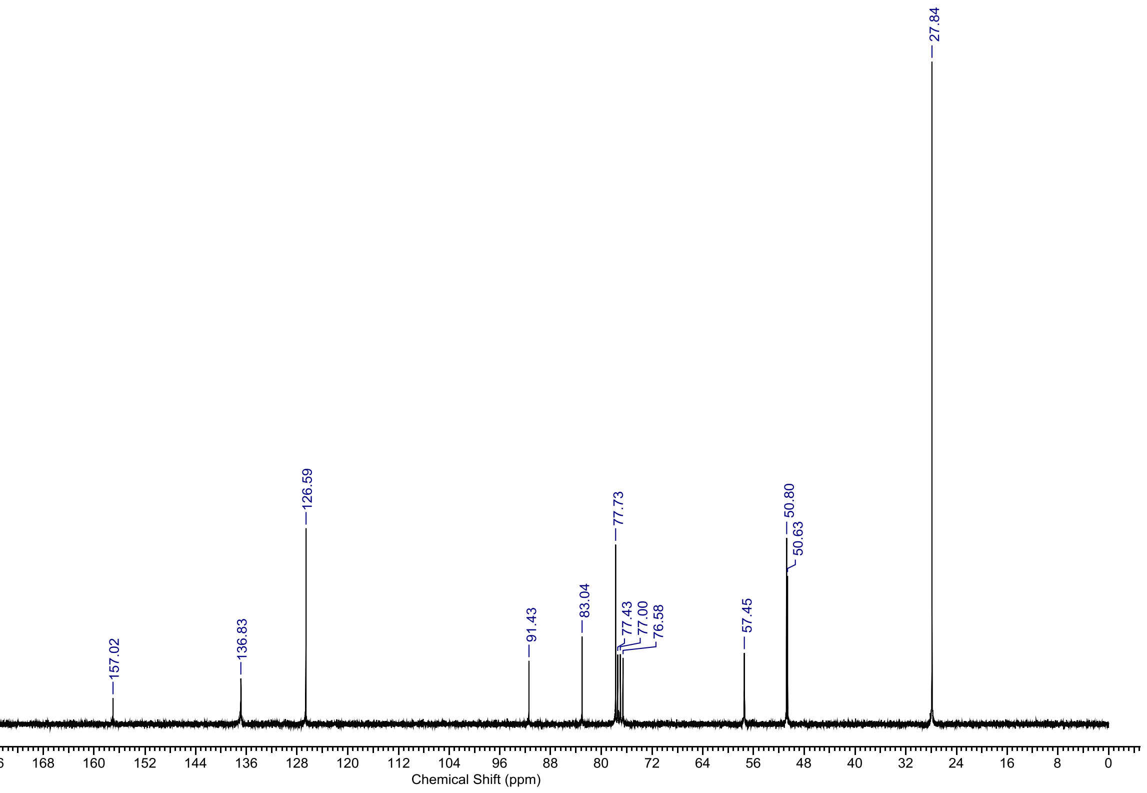

Supplement: File 1 — Experimental section. [file Beilstein_J_Org_Chem-14-531-s001.pdf]
